# Synthesis and evaluation of the antioxidant activity of new spiro-1,2,4-triazine derivatives applying Ag/Fe_3_O_4_/CdO@MWCNT MNCs as efficient organometallic nanocatalysts

**DOI:** 10.3389/fchem.2022.1001707

**Published:** 2022-09-29

**Authors:** Elham Ezzatzadeh, Somayeh Soleimani-Amiri, Zinatossadat Hossaini, Khatereh Khandan Barani

**Affiliations:** ^1^ Department of Chemistry, Ardabil Branch, Islamic Azad University, Ardabil, Iran; ^2^ Department of Chemistry, Karaj Branch, Islamic Azad University, Karaj, Iran; ^3^ Department of Chemistry, Qaemshahr Branch, Islamic Azad University, Qaemshahr, Iran; ^4^ Department of Chemistry, Zahedan Branch, Islamic Azad University, Zahedan, Iran

**Keywords:** Ag/Fe_3_O_4_/CdO@MWCNTs MNCs, spiro-1,2,4-triazines, antioxidant activity, ninhydrin, isatin, acenaphthene

## Abstract

We applied the *Petasites hybridus* rhizome water extract as green media so that Ag/Fe_3_O_4_/CdO@ multi-walled carbon nanotubes magnetic nanocomposites (Ag/Fe_3_O_4_/CdO@MWCNTs MNCs) could be prepared. We also evaluated its activity by using in the one-pot multicomponent reaction of acetophenones, diethyl oxalate, ammonium acetate, and activated carbonyl compounds such as ninhydrin, isatin and acenaphthylene-1,2-dione, and malononitrile and hydrazoyl chlorides in an aqueous medium at room temperature for the generation of spiro-1,2,4-triazines as new derivatives with tremendous output. Moreover, reducing organic pollutants from 4-nitrophenol (4-NP) was carried out by generating Ag/Fe_3_O_4_/CdO@MWCNTs in water at room temperature. The results displayed that Ag/Fe_3_O_4_/CdO@MWCNTs reduced pollutants of organic compounds in a short time. The synthesized spiro-1,2,4-triazines have NH and OH functional groups having acidic hydrogen with high antioxidant power. Also, the spiro-1,2,4-triazines exhibited antimicrobial ability. For this purpose, the disk diffusion method was applied and two kinds of bacteria, Gram-positive and Gram-negative, were employed for the analysis. Furthermore, we applied functional theory-based quantum chemical methods in order to better understand reaction mechanism density. To generate spiro-1,2,4-triazines, the applied process showed many properties such as reactions with short time, products with good yields, and simple extraction of catalyst from a mixture of reactions.

## Introduction

**SCHEME 1:**
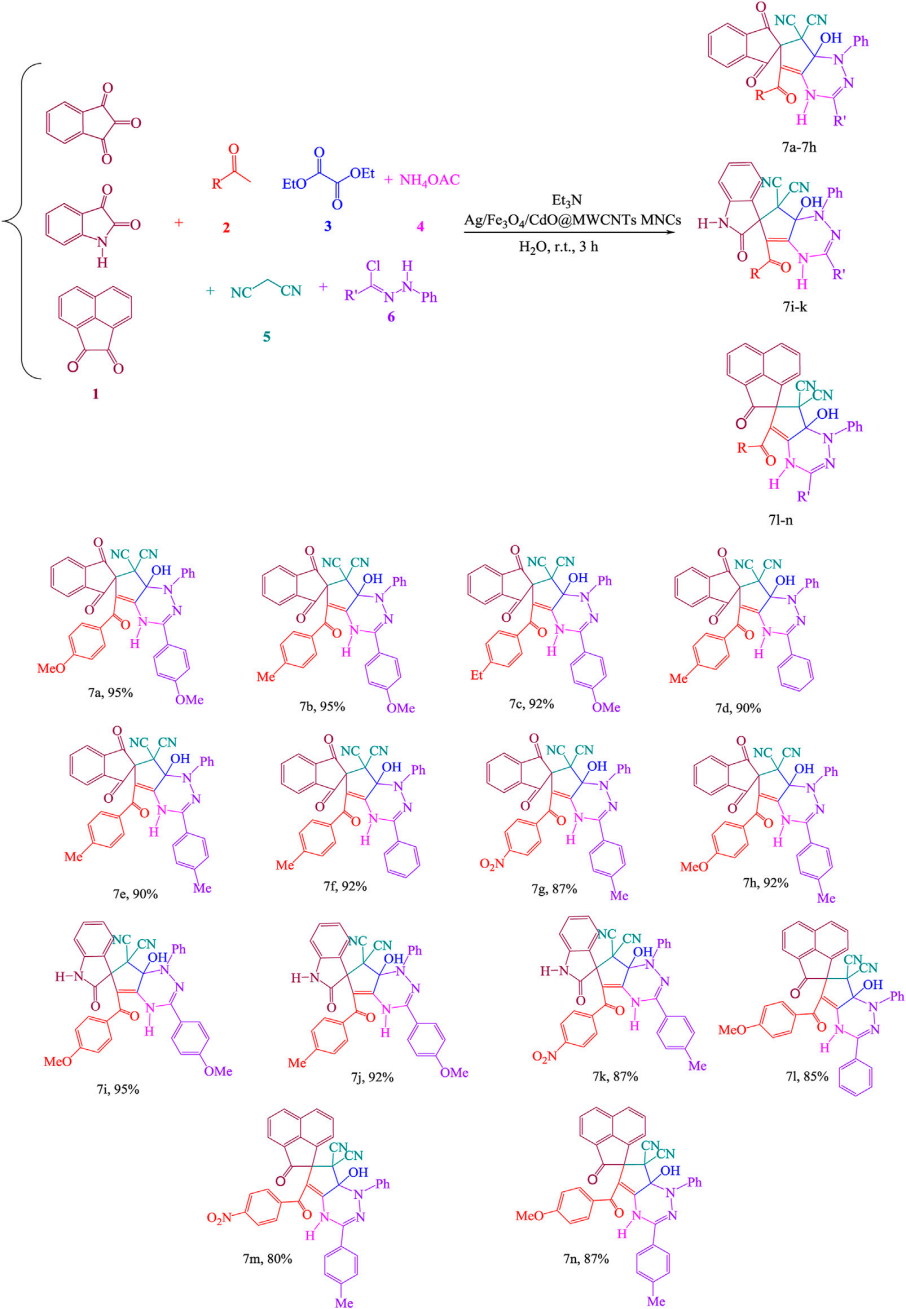
Synthesis of functionalized spiro-1,2,4-triazine **7**.

Among organic compounds, heterocyclic organic compounds are important because of their application in medicinal chemistry and having many biological activities (Goel et al., 2004; Amir et al., 2007; Siddiqui et al., 2011; Lamberth and Dinges, 2012; Abdolmohammadi et al., 2013; Abdolmohammadi 2014; Martins et al., 2015; Desai et al., 2016; Zhao et al., 2017; Fouad et al., 2018; Kalaria et al., 2018; Khattab and Rehan, 2018; Li et al., 2018; Sokolova et al., 2018). Thus, due to the importance of these compounds, many procedures have been reported for the synthesis of heterocyclic compounds. One common procedure to synthesize these compounds with biological activity is multicomponent reactions (MCRs) (Ibarra et al., 2018; Zhi et al., 2019; Borah et al., 2021; Kumar et al., 2022). MCRs are significant due to having benefits such as atom effectiveness and synthesis of heterocyclic compounds with high yields compared with other procedures for the synthesis of heterocyclic compounds (Weber et al., 1999; Tietze et al., 2006; Herrera and Marqués-López, 2015; Borah, et al., 2021). In some procedures for the synthesis of heterocyclic compounds, a catalyst is needed. The transition metal oxide nanostructures with a high active surface area could be used as a catalyst in these reactions. Also, these catalysts are employed in technology and applied science (Sahay et al., 2012; Zhao et al., 2019; Chen et al., 2020). MWCNTs have been widely investigated, due to their large surface area and high adsorption ability (Zhang et al., 2013; Abdolmohammadi 2018; Abdolmohammadi and Hossaini., 2019; Abdolmohammadi et al., 2020). Recently, the supported catalyst and bimetallic oxide or trimetallic oxide catalysts have drawn attention owing to their high capabilities to carry out high-selectivity and efficient organic reactions (Wachs 2005; Dastan et al., 2012; Guo et al., 2014; Janitabar-Darzi and Abdolmohammadi, 2019; Abdolmohammadi et al., 2022). Metal oxides possess great crystalline structure and catalytic efficiency (Shi 2013; Jablonska and Palkovits, 2016). This is the reason why the mixture of two or more metals and their curing mechanisms allow the change in the properties of material surfaces for the optimization of the properties for a particular goal (Zhang et al., 2012; Lin-Bing et al., 2015). Accordingly, the combination of metal oxide catalysts and their nanocomposite structure has exhibited the production of heterocyclic compounds according to green rules with high efficiency (Kalantari et al., 2020; Abdolmohammadi et al., 2021; Khalilzadeh et al., 2021). Among the metal oxide nanoparticles, Fe_3_O_4_ magnetic nanoparticles (MNPs) are important because of their high surface area, simply removed from reaction, and their application in MCRs several times. Another subject addressed by this research was biological abilities such as the antioxidant and antimicrobial activity of synthesized spiro pyridoindole pyrrolidines. Compounds with antioxidant activity could eliminate the negative effect of free radicals due to having a reduced chemical structure. Also, these compounds could be employed as transitional metals chelators and they have been effective in improving or treating many illnesses (Halliwell 1999; Liu and Meydani, 2002; Babizhayev et al., 2004; Ahmadi et al., 2007; Djurišić et al., 2012). Another area of investigation on biological activity is the antimicrobial power of synthesized compounds. Some bacteria cannot be killed by utilizing drugs and they cause many diseases in humans and animals. For this reason, it is of great significance to find out procedures with good yield for decreasing this problem and investigating the antimicrobial properties of synthesized compounds. Dyes and pigments are two important components that are used in generating processes of food, drug, textile, and print. Producing dyes and pigments amount to about ∼7 × 105 tons in 1 year, which is hazardous for aquatic system organisms (Mandel et al., 2013). For this reason, discovering green and eco-friendly procedures for removing dyes and pigment pollutants from the environment is very important. Most of the procedures that have been reported in the literature have used much energy and generated adverse by-products. Therefore, high-efficiency methods or active synthesized compounds are required for eliminating or decreasing these problems. Over recent years, enhancing new and easy processes for the generation of essential heterocyclic compounds have been dealt with (Yavari et al., 2007; Yavari et al., 2008; Yavari et al., 2009; Yavari et al., 2010; Hajinasiri et al., 2011; Rostami Charati et al., 2011; Rustaiyan, and Ezzatzadeh, 2011; Ezzatzadeh et al., 2012; Tavakolinia et al., 2012; Hossaini et al., 2014; Rajabi et al., 2015; Rostami-Charati et al., 2015; Rezayati et al., 2016; Sajjadi-Ghotbabadi et al., 2016; Rostami-charati et al., 2017a; Rostami-Charati et al., 2017b; Hossaini et al., 2017; Ezzatzadeh 2018; Rezayati et al., 2018; Ezzatzadeh and Hossaini, 2019a; Ezzatzadeh and Hossaini 2019b; Seifi Mansour et al., 2019; Ezzatzadeh and Hossaini 2020a; Aghahosseini, and Ramazani, 2020; Ahmadi et al., 2020; Baghernejad and Fiuzat 2020; Ezzatzadeh and Hossaini, 2020b; Ebrahimi et al., 2020; Ghanaat et al., 2020; Haddazadeh and Mohammadi, 2020; Hakimi et al., 2020; Khazaei et al., 2020; Mostaghni and Taat, 2020; Nikpassand and Zare Fekri, 2020; Raoufi et al., 2020; Rohaniyan et al., 2020; Sajjadifar et al., 2020; Taran et al., 2020; Tayebee and Gohari, 2020; Baghernejad and Nazari, 2021; Baghernejad and Rostami Harzevili, 2021; Kamali and Shirini, 2021; Karimi et al., 2021; Mohammed Abd Al-Mohson 2021; Salih and Al-Messri, 2021). In this research, initially, a green procedure was employed for the generation of new spiro-1,2,4-triazine **7** *via* MCRs of activated carbonyl compound **1**, acetophenones **2**, diethyl oxalate **3**, ammonium acetate **4**, malononitrile **5**, and hydrazoyl chloride **6** in the aqueous form at room temperature in the vicinity of Ag/Fe_3_O_4_/CdO@MWCNT MNCs as an organometallic catalyst in aqueous media at room temperature (Scheme 1).

## Experimental

### General

In this research work, all of the starting materials needed for the synthesis of spiro-1,2,4-triazines and also reagents and solvents were prepared by Fluka and Merck Company with no further purification. For the synthesis of the nanocatalyst, the MWCNTs were used with 8 nm for diameter, 30 μm ling, and 95% of purity, prepared by Merck Company. For approving the construction of synthesized catalyst Ag/Fe_3_O_4_/CdO@MWCNT MNCs, spectroscopy analysis such as XRD, SEM, EDX, and VSM was utilized. The FT-IR (KBr medium) of synthesized spiro-1,2,4-triazine was prepared by Shimadzu IR-460 spectrometer instrument. Additionally, another way to confirm the makeup of synthesized compounds was by applying 1H-NMR and 13C-NMR with Bruker DRX-500 AVANCE spectrometer instrument with 500 MHz NMR in CDCl3 as solvent and TMS as the internal standard. Mass spectra for synthesized compounds were given by Finnigan, MAT 8430 spectrometer with an ionization potential of 70 eV. To determine the element in the prepared compounds, we used a Heraeus CHN–O-Rapid analyzer.

### Producing *Petasites hybridus* rhizome water extract

After drying the *Petasites hybridus* rhizome, 10 g of it was poured into a two-neck round bottom flask (250 ml), followed by adding water (100 ml) and stirring the new mixture at 100°C and filtered after 2 h. For the production of the nanocomposite, the water extract of *Petasites hybridus* rhizome and other compounds were employed as follows:

### Generation of Ag/Fe_3_O_4_/CdO@MWCNT MNCs

After dissolving the mixture of Cd (NO_3_)_2_ (1.5 g) and FeCl_2_.4H_2_O (1.5 g) in water (10 ml), the next step was to add the *Petasites hybridus* rhizome water extract (5 ml) to the previous mixture, and the temperature of the mixture was enhanced to 100°C in a round bottom flask and mixed for 5 h. When the reaction was completed, the temperature of the reaction was reduced to room temperature. Next, after cooling, to remove the unwanted organic compounds, the mixture experienced sonication for 30 min and was centrifuged at 7,000 rpm for about 10 min. Then, we poured AgNO3 (1.5 g) into the previous mixture and continued the sonication of the new mixture at 100°C for 45 min, and thus, Ag//Fe_3_O_4_/CdO MNCs were synthesized. To synthesize Ag//Fe_3_O_4_/CdO@MWCNTs MNCs, the MWCNTs (0.1 g) and prepared Ag//Fe_3_O_4_/CdO MNCs (0.1 g) in the previous section were added to 100 ml water extract of *Petasites hybridus* rhizome and mixed at 150°C for 1 h. We used a centrifuge to separate the colloid, then we washed it with water, dried it, and calcinated it at 300°C for 45 min. After this, the Ag//Fe_3_O_4_/CdO @MWCNT magnetic nanocomposite was produced, which needed to be cooled to room temperature and washed with a mixture of water and ethanol (50:50) several times. After washing the solid, by employing an external magnet, the catalyst was separated and dried at room temperature for 24 h (Rajendran and Sengodan, 2017).

### Preparation process of spiro-1,2,4-triazines 7a–n

Acetophenones **2** (2 mmol), diethyl oxalate **3** (2 mmol), and Ag//Fe_3_O_4_/CdO@MWCNT (0.02 g) were added to water as solvent at room temperature and mixed for 30 min. We poured ammonium acetate **4** (2 mmol) into the previous mixture after 30 min and mixed the new mixture for 30 min, followed by adding activated carbonyl compound **1** (2 mmol) and malononitrile **5** (2 mmol) together into another pot in the presence of the catalyst for 45 min. It was then added to the previous mixture and stirred for 30 min. Finally, hydrazoyl chloride **6** (2 mmol) was added and the ultimate mixture was stirred for 1 h in the presence of the catalyst. The reaction was accomplished after 3 h and it was monitored by TLC; in this stage, the separation took place using an external magnet, and the solid residue was washed by EtOH and Et2O to prepare purified spiro-1,2,4-triazines **7**.

7a-Hydroxy-5-(4-methoxybenzoyl)-3- (4-methoxyphenyl)-1′,3′-dioxo-1-phenyl-1,1′,3′,7a- tetrahydrospiro[cyclopenta[e][1,2,4]triazine-6,2′-indene]-7,7(4H)-dicarbonitrile **(7a)**: yellow powder, m.p. 138–140°C. Yield: 95%. IR (KBr) (ν_max_/cm^−1^): 3,565, 3,348, 2,195, 1,728, 1,725, 1,697, 1,485, 1,378, and 1,292 cm^−1^. ^1^H NMR (500 MHz, CDCl_3_): δ ppm: 3.85 (3 H, s, OMe), 3.78 (3 H, s, OMe), 6.56 (1 H, s, OH), 6.95 (2 H, d, ^3^J = 7.7 Hz, 2 CH), 7.18–7.23 (4 H, m, 4 CH), 7.28–7.32 (3 H, m, 3 CH), 7.35–7.42 (4 H, m, 4 CH), 7.52–7.57 (3 H, m, 3 CH), 7.87 (2 H, d, ^3^J = 7.7 Hz, 2 CH), and 10.35 (1 H, s, NH) ppm. ^13^C NMR (125.7 MHz, CDCl_3_): δ 50.9, 55.3, 67.9, 95.4, 112.7, 114.7, 118.7, 122.7, 123.7, 126.4, 128.4, 128.8, 129.3, 131.6, 130.2, 133.7, 135.4, 137.0, 137.0, 138.8, 138.8, 140.4, 163.9, 191.7,198.5, and 198.7. MS, m/z (%): 605 (M^+^, 10), 147 (48), and 31 (100). Anal. Calcd for C_36_H_23_N_5_O_5_ (605.61): C, 71.40; H, 3.83; and N, 11.56; found: C, 71.35; H, 3.78; N, and 11.48%.

7a-Hydroxy-3-(4-methoxyphenyl)-5-(4-methyl benzoyl)-1′,3′-dioxo-1-phenyl-1,1′,3′,7a- tetrahydrospiro[cyclopenta[e][1,2,4]triazine-6,2′-indene]-7,7(4H)-dicarbonitrile **(7b)**: yellow powder, m.p. 143–145°C. Yield: 0.96 g (95%). IR (KBr) (ν_max_/cm^−1^): 3,456, 3,357, 2,232, 1,729, 1,727, 1,698, 1,589, 1,487, 1,358, and 1,296 cm^−1^. ^1^H NMR (500 MHz, CDCl_3_): δ ppm: 2.25 (3 H, s, Me), 3.86 (3 H, s, OMe), 6.57 (1 H, s, OH), 6.85 (2 H, d, ^3^J = 7.7 Hz, 2 CH), 7.16 (2 H, d, ^3^J = 7.7 Hz, 2 CH), 7.25–7.29 (3 H, m, 3 CH), 7.32–7.37 (4 H, m, 4 CH), 7.42 (2 H, m, 2 CH), 7.45 (2 H, d, ^3^J = 7.8 Hz, 2 CH), 7.63 (2 H, d, ^3^J = 7.8 Hz, 2 CH), and 10.45 (1 H, s, NH). ^13^C NMR (125.7 MHz, CDCl_3_): δ 21.3, 50.9, 55.3, 67.9, 95.4, 114.1,114.7, 118.6, 122.6, 123.6, 126.4, 126.9, 127.7, 128.3,129.2, 129.4, 135.4, 136.4, 137.5, 137.8, 138.5, 138.9, 140.4, 142.9, 161.2, 191.9, 198.5, and 198.5. MS, m/z (%): 619 (M^+^, 10), 147 (48), and 31 (100). Anal. Calcd for C_37_H_25_N_5_O_5_ (619.64): C, 71.72; H, 4.07; and N, 11.30; found: C, 71.78; H, 4.12; and N, 11.36; %.

5-(4-Ethyl benzoyl)-7a-hydroxy-3-(4-methoxyphenyl)-1′,3′-dioxo-1-phenyl-1,1′,3′,7a- tetrahydrospiro[cyclopenta[e][1,2,4]triazine-6,2′-indene]-7,7(4H)-dicarbonitrile **(7c)**: pale yellow powder, m.p. 153–154°C. Yield: 92%. IR (KBr) (ν_max_/cm^−1^): 3,467, 3,348, 2,175, 1,732, 1,727, 1,996, 1,586, 1,485, 1,368, and 1,297 cm^−1^. ^1^H NMR (500 MHz, CDCl_3_): δ ppm: 1.35 (3 H, t, ^3^J_HH_ = 7.4 Hz, Me), 2.75 H, q, ^3^J_HH_ = 7.4 Hz, CH_2_), 3.78 (3 H, s, OMe), 6.63 (1 H, s, OH), 6.94 (2 H, d, ^3^J = 7.8 Hz, 2 CH), 7.16 (2 H, d, ^3^J = 7.8 Hz, 2 CH), 7.23 (2 H, d, ^3^J = 7.8 Hz, 2 CH), 7.26–7.29 (3 H, m, 3 CH), 7.34–7.36 (3 H, m, 3 CH), 7.42–7.45 (4 H, m, 4 CH), 7.52 (2 H, d, ^3^J = 7.8 Hz, 2 CH), and 10.48 (1 H, s, NH). ^13^C NMR (125.7 MHz, CDCl_3_): 15.3, 28.6, 50.9, 55.3, 67.9, 95.4, 114.1, 114.7, 118.6, 122.6, 123.6, 126.4, 126.9, 127.5, 127.7, 129.3, 129.4, 135.4, 136.3, 137.2, 137.5, 138.5, 138.8, 140.4, 146.8, 161.2, 191.8, 197.3, and 198.5. MS, m/z (%): 633 (M^+^, 10), 147 (48), and 31 (100). Anal. Calcd for C_38_H_27_N_5_O_5_ (633.66): C, 72.03; H, 4.30; and N, 11.05; found: C, 72.12; H, 4.39; and N, 11.12%.

7a-Hydroxy-5-(4-methyl benzoyl)-1′,3′-dioxo-1,3-diphenyl-1,1′,3′,7a-tetrahydrospiro [cyclopenta[e][1,2,4]triazine-6,2′-indene]-7,7(4H)-dicarbonitrile **(7d)**: yellow powder, m.p. 152–154°C. Yield: 90%. IR (KBr) (ν_max_/cm^−1^): 3,478, 3,365, 2,195, 1,734, 1,728, 1,698, 1,595, 1,485, 1,376, and 1,293 cm^−1^. ^1^H NMR (500 MHz, CDCl3): δ ppm: 2.36 (3 H, s, Me), 6.52 (1 H, s, OH), 6.85–7.03 (4 H, m, 4 CH), 7.12–7.16 (4 H, m, 4 CH), 7.25 (2 H, d, ^3^J_HH_ = 7.6 Hz, 2 CH), 7.28–7.32 (3 H, m, 3 CH), 7.42–7.47 (3 H, m, 3 CH), 7.56 (2 H, d, ^3^J = 7.8 Hz, 2 CH), and 10.68 (1 H, s, NH). ^13^C NMR (125.7 MHz, CDCl_3_): δ 21.3, 50.9, 67.6, 95.4, 114.7, 118.6, 122.7, 123.6, 126.4, 127.7, 128.2, 129.2, 130.4, 131.2, 132.3, 133.2, 135.4, 136.4, 137.2, 137.8, 138.8, 139.3, 140.4, 142.9, 191.9, 197.6, and 198.5. MS, m/z (%): 589 (M^+^, 10), 147 (48), and 31 (100). Anal. Calcd for C_36_H_23_N_5_O_4_ (589.61): C, 73.34; H, 3.93; and N, 11.88; found: C, 73.42; H, 4.02; and N, 11.93%.

7a-Hydroxy-5-(4-methyl benzoyl)-1′,3′-dioxo-1-phenyl-3-(p-tolyl)-1,1′,3′,7a-tetrahydrospiro [cyclopenta[e][1,2,4]triazine-6,2′-indene]-7,7(4H)-dicarbonitrile **(7e)**: yellow powder, m.p. 163–165°C. Yield: 90%. IR (KBr) (ν_max_/cm^−1^): 3,546, 3,384, 1,732, 1,725, 1,695, 1,588, 1,498, 1,378, and 1,295 cm^−1^. 1H NMR (500 MHz, CDCl_3_): δ ppm: 2.35 (3 H, s, Me), 2.43 (3 H, s, Me), 6.38 (1 H, s, OH), 6.94 (2 H, d, ^3^J = 7.6 Hz, 2 CH), 7.02 (2 H, d, ^3^J_HH_ = 7.6 Hz, 2 CH), 7.06–7.09 (3 H, m, 3 CH), 7.18–7.24 (4 H, m, 4 CH), 7.36 (2 H, d, ^3^J = 7.8 Hz, 2 CH), 7.42 (2 H, d, ^3^J = 7.6 Hz, 2 CH), 7.45–7.47 (2 H, m, 2 CH), and 10.45 (1 H, s, NH). ^13^C NMR (125.7 MHz, CDCl_3_): δ 21.2, 21.3, 50.9, 67.9, 95.3, 114.7, 118.6, 122.6, 123.6, 124.5, 125.3, 126.4, 127.5, 128.3, 129.2, 129.6, 135.4, 136.3, 137.0, 137.6, 138.7, 138.8, 140.4, 141.4, 142.9, 191.9, 198.4, and 198.5. MS, m/z (%): 603 (M^+^, 10), 147 (48), and 31 (100). Anal. Calcd for C_37_H_25_N_5_O_4_ (603.64): C, 73.34; H, 3.93; and N, 11.88; found: C, 73.42; H, 4.02; and N, 11.93%.

7a-Hydroxy-5-(4-methoxybenzoyl)-1′,3′- dioxo-1,3-diphenyl-1,1′,3′,7a-tetrahydrospiro [cyclopenta[e][1,2,4]triazine-6,2′-indene]-7,7(4H)-dicarbonitrile **(7f)**: yellow powder, m.p. 171–173°C. Yield: 92%. IR (KBr) (ν_max_/cm^−1^): 3,547, 3,368, 1,734, 1,726, 1,697, 1,597, 1,488, 1,387, and 1,292 cm^−1^. ^1^H NMR (500 MHz, CDCl_3_): δ ppm: 2.38 (3 H, s, Me), 6.57 (1 H, s, OH), 6.92 (2 H, d, ^3^J = 7.8 Hz, 2 CH), 7.05–7.08 (3 H, m, 3 CH), 7.12–7.16 (4 H, m, 4 CH), 7.23–7.28 (4 H, m, 4 CH), 7.34 (2 H, d, ^3^J = 7.8 Hz, 2 CH), 7.37–7.42 (3 H, m, 3 CH), and 10.84 (1 H, s, NH). ^13^C NMR (125.7 MHz, CDCl_3_): δ 21.3, 50.9, 67.9, 95.3, 114.7, 118.6, 122.6, 123.6, 126.4, 127.7, 128.2, 128.3, 128.9, 129.2, 130.1, 131.5, 135.4, 136.3, 137.0, 137.04, 138.8, 138.9, 140.4, 142.9, 191.9, 198.4, and 198.5. MS, m/z (%): 589 (M^+^, 10), 147 (48), and 31 (100). Anal. Calcd for C_36_H_23_N_5_O_4_ (589.61): C, 73.34; H, 3.93; and N, 11.88; found: C, C, 73.43; H, 4.06; and N, 11.95%.

7a-Hydroxy-5-(4-nitrobenzoyl)-1′,3′- dioxo-1-phenyl-3-(p-tolyl)-1,1′,3′,7a-tetrahydrospiro [cyclopenta[e][1,2,4]triazine-6,2′-indene]-7,7(4H)-dicarbonitrile **(7g)**: yellow powder, m.p. 172–174°C. Yield: 87%. IR (KBr) (ν_max_/cm^−1^): 3,452, 3,365, 1,729, 1,726, 1,694, 1,584, 1,469, and 1,297 cm^−1^. ^1^H NMR (500 MHz, CDCl3): δ ppm: 2.47 (3 H, s, Me), 6.58 (1 H, s, OH), 6.92 (2 H, d, ^3^J = 7.7 Hz, 2 CH), 7.06–7.11 (4 H, m, 4 CH), 7.26 (2 H, d, ^3^J = 7.7 Hz, 2 CH), 7.34–7.37 (2 H, m, 2 CH), 7.45–7.48 (3 H, m, 3CH), 7.53 (2 H, d, ^3^J = 7.7 Hz, 2 CH), 8.06 (2 H, d, ^3^J = 7.8 Hz, 2 CH), and 10.24 (1 H, s, NH). ^13^C NMR (125.7 MHz, CDCl_3_): δ 21.3, 50.9, 67.9, 95.3, 114.7, 118.6, 121.4, 122.6, 123.6, 126.4, 128.3, 129.2, 130.4, 132.5, 133.2, 135.4, 137.2, 137.8, 138.7, 138.8, 139.2, 140.8, 141.3, 149.0, 191.5, 198.4, and 198.5. MS, m/z (%): 634 (M^+^, 10), 147 (48), and 31 (100). Anal. Calcd for C_36_H_22_N_6_O_6_ (634.61): C, 68.14; H, 3.49; and N, 13.24; found: C, 68.23; H, 3.58; and N, 13.42%.

7a-Hydroxy-5-(4-methoxybenzoyl)-1′,3′-dioxo-1-phenyl-3-(p-tolyl)-1,1′,3′,7a-tetrahydrospiro [cyclopenta[e][1,2,4]triazine-6,2′-indene]-7,7(4H)-dicarbonitrile **(7h)**: pale yellow powder, m.p. 149–151°C. Yield: 92%. IR (KBr) (ν_max_/cm^−1^): 3,458, 3,364, 1,732, 1,726, 1,698, 1,578, 1,364, and 1,286 cm^−1^. ^1^H NMR (500 MHz, CDCl3): δ ppm: 2.37 (3 H, s, Me), 3.87 (3 H, s, OMe), 6.62 (1 H, s, OH), 6.93 (2 H, d, ^3^J = 7.7 Hz, 2 CH), 7.14 (2 H, d, ^3^J = 7.7 Hz, 2 CH), 7.22–7.26 (3 H, m, 3 CH), 7.30–7.35 (4 H, m, 4 CH), 7.38 (2 H, m, 2 CH), 7.43 (2 H, d, ^3^J = 7.8 Hz, 2 CH), 7.56 (2 H, d, ^3^J = 7.8 Hz, 2 CH), and 10.63 (1 H, s, NH). ^13^C NMR (125.7 MHz, CDCl3): δ 21.5, 51.3, 55.6, 68.3, 95.7, 114.6, 115.3, 118.5, 123.4, 124.5, 127.2, 128.3, 129.5, 130.2, 131.2, 132.3, 134.2, 135.3, 136.2, 137.5, 138.6, 139.3, 141.2, 143.2, 162.3, 192.4, 197.2, and 198.6. MS, m/z (%): 619 (M^+^, 10), 147 (48), and 31 (100). Anal. Calcd for C_37_H_25_N_5_O_5_ (619.64): C, 71.72; H, 4.07; and N, 11.30; found: C, 71.78; H, 4.12; and N, 11.36; %.

7a-Hydroxy-5-(4-methoxybenzoyl)-3-(4-methoxyphenyl)-2′-oxo-1-phenyl-1,7a-dihydrospiro[cyclopenta[e][1,2,4]triazine-6,3′-indoline]-7,7(4H)-dicarbonitrile **(7i)**: yellow powder, m.p. 179–181°C. Yield: 1.01 g (92%). IR (KBr) (ν_max_/cm^−1^): 3,478, 3,374, 1,697, 1,732, 1,727, 1,595, 1,478, 1,376, and 1,284 cm^−1^. ^1^H NMR (500 MHz, CDCl_3_): δ ppm: 3.78 (3 H, s, OMe), 3.85 (3 H, s, OMe), 6.43 (1 H, s, OH), 6.89 (2 H, d, ^3^J = 7.7 Hz, 2 CH), 7.02 (2 H, d, ^3^J = 7.8 Hz, 2 CH), 7.05–7.09 (4 H, m, 4 CH), 7.17–7.22 (3 H, m, 3 CH), 7.32 (2 H, d, ^3^J = 7.7 Hz, 2 CH), 7.38 (2 H, d, ^3^J = 7.8 Hz, 2 CH), 7.42–7.45 (2 H, m, 2 CH), 9.87 (1 H, s, NH), and 10.25 (1 H, s, NH). ^13^C NMR (125.7 MHz, CDCl_3_): δ 55.2, 55.3, 55.3, 66.9, 94.9, 111.5, 112.7, 113.8, 114.2, 118.6, 122.5,123.7,124.4,124.9,126.7, 127.4, 128.6, 129.3, 130.4, 131.2, 133.6, 138.4, 139.2, 140.3, 141.2, 161.0, 163.9, 175.8, and 192.2. MS, m/z (%): 619 (M^+^, 10), 147 (48), and 31 (100). Anal. Calcd for C_37_H_26_N_6_O_5_ (622.64): C, 69.45; H, 4.21; and N, 13.50; found: C, 69.53; H, 4.32; and N, 13.65%.

7a-Hydroxy-3-(4-methoxyphenyl)-5- (4-methylbenzoyl)-2′-oxo-1-phenyl-1,7a-dihydrospiro[cyclopenta [e][1,2,4]triazine-6,3′-indoline]-7,7(4H)-dicarbonitrile **(7j)**: yellow powder, m.p. 182–184°C. Yield: 87%. IR (KBr) (ν_max_/cm^−1^): 3,487, 3,369, 2,195, 1,735, 1,728, 1,689, 1,587, 1,486, 1,387, and 1,268 cm^−1^. ^1^H NMR (500 MHz, CDCl_3_): δ ppm: 2.32 (3 H, s, Me), 3.89 (3 H, s, OMe), 6.63 (1 H, s, OH), 6.93 (2 H, d, ^3^J = 7.7 Hz, 2 CH), 7.13 (2 H, d, ^3^J = 7.7 Hz, 2 CH), 7.19–7.24 (3 H, m, 3 CH), 7.28–7.33 (4 H, m, 4 CH), 7.38–7.42 (2 H, m, 2 CH), 7.47 (2 H, d, ^3^J = 7.8 Hz, 2 CH), 7.54 (2 H, d, ^3^J = 7.8 Hz, 2 CH), 9.85 (1 H, s, NH), and 10.67 (1 H, s, NH). ^13^C NMR (125.7 MHz, CDCl_3_): δ 21.6, 51.3, 55.7, 68.3, 96.3, 113.7, 114.2, 118.7, 121.8, 123.4, 125.6, 126.5, 127.4, 128.2, 129.7, 130.4, 134.8, 136.5, 137.2, 138.4, 139.3, 140.7, 141.2, 142.7, 161.4, 163.4, and 191.7. MS, m/z (%): 606 (M^+^, 10), 147 (48), and 31 (100). Anal. Calcd for C_36_H_26_N_6_O_4_ (606.64): C, 71.28; H, 4.32; and N, 13.85; found: C, 71.36; H, 4.43; and N, 13.97%.

7a-Hydroxy-5-(4-nitrobenzoyl)-2′-oxo-1,3 -diphenyl-1,7a-dihydrospiro[cyclopenta[e] [1,2,4]triazine-6,3′-indoline]-7,7(4H)-dicarbonitrile **(7k)**: yellow powder, m.p. 192–194°C. Yield: 87%. IR (KBr) (ν_max_/cm^−1^): 3,458, 3,345, 2,197, 1,736, 1,727, 1,695, 1,587, 1,463, 1,357, and 1,296 cm^−1^. ^1^H NMR (500 MHz, CDCl_3_): δ ppm: 6.67 (1 H, s, OH), 6.95 (2 H, d, ^3^J = 7.8 Hz, 2 CH), 7.04–7.08 (4 H, m, 4 CH), 7.18 (2 H, d, ^3^J = 7.7 Hz, 2 CH), 7.22–7.25 (2 H, m, 2 CH), 7.33–7.37 (3 H, m, 3CH), 7.48 (2 H, d, ^3^J = 7.7 Hz, 2 CH), 8.06 (2 H, d, ^3^J = 7.8 Hz, 2 CH), 9.68 (1 H, s, NH), and 10.37 (1 H, s, NH). ^13^C NMR (125.7 MHz, CDCl_3_): δ 21.7, 51.3, 68.3, 95.5, 115.8, 119.3, 121.7, 122.5, 123.7, 126.6, 128.7, 129.5, 131.2, 132.7, 133.5, 135.6, 136.8, 137.5, 138.3, 139.3, 140.5, 141.3, 142.4, 148.4, 164.3, and 191.7. MS, m/z (%): 621 (M^+^, 10), 147 (48), and 31 (100). Anal. Calcd for C_35_H_23_N_7_O_5_ (621.61): C, 67.63; H, 3.73; and N, 15.77; found: C, 67.74; H, 3.86; and N, 15.92%.

7a′-Hydroxy-5′-(4-methoxybenzoyl)-2-oxo-1′,3′- diphenyl-1′,7a′-dihydro-2H-spiro [acenaphthylene-1,6′-cyclopenta[e][1,2,4]triazine]-7′,7′(4′H)-dicarbonitrile **(7l)**: yellow powder, m.p. 186–188°C. Yield: 85%. IR (KBr) (ν_max_/cm^−1^): 3,574, 3,358, 2,185, 1,735, 1,727, 1,694, 1,489, 1,368, and 1,297 cm^−1^. ^1^H NMR (500 MHz, CDCl_3_): δ ppm: 3.78 (3 H, s, OMe), 6.82 (1 H, s, OH), 6.97 (2 H, d, ^3^J = 7.8 Hz, 2 CH), 7.03–7.07 (5 H, m, 5 CH), 7.12–7.16 (3 H, m, 3 CH), 7.23–7.27 (4 H, m, 4 CH), 7.38 (2 H, d, ^3^J = 7.8 Hz, 2 CH), 7.43–7.47 (4 H, m, 4 CH), and 10.37 (1 H, s, NH). ^13^C NMR (125.7 MHz, CDCl_3_): δ 55.3, 56.7, 66.5, 95.4, 112.7, 113.4, 114.5, 115.2, 116.3, 117.6, 118.5, 119.4, 120.3, 121.8, 122.3, 122.8, 123.4, 124.5, 125.3, 126.7, 128.5, 127.2, 129.3, 130.8, 131.6, 132.4, 133.2, 136.5, 137.4, 138.5, 139.2, 140.3, 163.9, 192.2, and 195.3. MS, m/z (%): 627 (M+, 10), 147 (48), and 31 (100). Anal. Calcd for C_39_H_25_N_5_O_4_ (627.66): C, 74.63; H, 4.01; and N, 11.16; found: C, 74.73; H, 4.14; and N, 11.28%.

7a′-Hydroxy-5′-(4-nitrobenzoyl)-2-oxo-1′-phenyl-3′-(p-tolyl)-1′,7a′-dihydro-2H-spiro[acenaphthylene-1,6′- cyclopenta[e][1,2,4]triazine]-7′,7′(4′H)-dicarbonitrile **(7m)**: yellow powder, m.p. 193–195°C. Yield: 80%. IR (KBr) (ν_max_/cm^−1^): 3,547, 3,378, 2,186, 1,736, 1,726, 1,692, 1,568, 1,367, and 1,297 cm^−1^. ^1^H NMR (500 MHz, CDCl_3_): δ ppm: 2.42 (3 H, s, Me), 6.57 (1 H, s, OH), 6.95 (2 H, d, ^3^J = 7.7 Hz, 2 CH), 7.12 (2 H, d, ^3^J = 7.7 Hz, 2 CH), 7.16–7.21 (4 H, m, 4 CH), 7.27–7.32 (4 H, m, 4 CH), 7.38 (2 H, d, ^3^J = 7.7 Hz, 2 CH), 7.42–7.46 (3 H, m, 3 CH), 8.06 (2 H, d, ^3^J = 7.8 Hz, 2 CH), and 10.38 (1 H, s, NH). ^13^C NMR (125.7 MHz, CDCl_3_): δ 21.3, 56.7, 66.67, 95.5, 112.3, 113.6, 114.2, 115.8, 116.4, 117.6, 118.7, 119.2, 120.8, 121.5, 122.8, 123.5, 124.2, 125.7, 127.3, 128.5, 129.3, 129.7, 130.8, 132.4, 136.5, 137.4, 138.5, 139.3, 143.4, 145.3, 146.3, 149.2, 191.3, and 196.3. MS, m/z (%): 656 (M^+^, 10), 147 (48), and 31 (100). Anal. Calcd for C_39_H_24_N_6_O_5_ (656.66): C, 71.34; H, 3.68; and N, 12.80; found: C, 71.45; H, 3.78; and N, 12.92; %.

7a′-Hydroxy-5′-(4-methoxybenzoyl)-2-oxo-1′- phenyl-3′-(p-tolyl)-1′,7a′-dihydro-2H-spiro[acenaphthylene-1,6′- cyclopenta[e][1,2,4]triazine]-7′,7′(4′H)-dicarbonitrile **(7n)**: yellow powder, m.p. 181–183°C. Yield: 87%). IR (KBr) (ν_max_/cm^−1^): 3,486, 3,367, 2,234, 1,738, 1,728, 1,697, 1,587, 1,492, 1,387, and 1,265 cm^−1^. ^1^H NMR (500 MHz, CDCl_3_): δ ppm: 2.42 (3 H, s, Me), 3.85 (3 H, s, OMe), 6.57 (1 H, s, OH), 6.92 (2 H, d, ^3^J = 7.7 Hz, 2 CH), 7.12 (2 H, d, ^3^J_HH_ = 7.8 Hz, 2 CH), 7.14–7.17 (5 H, m, 5 CH), 7.23–7.27 (3 H, m, 3 CH), 7.38 (2 H, d, ^3^J = 7.8 Hz, 2 CH), 7.43–7.47 (3 H, m, 3 CH), 7.54 (2 H, d, ^3^J = 7.8 Hz, 2 CH), and 10.64 (1 H, s, NH). ^13^C NMR (125.7 MHz, CDCl_3_): δ 21.3, 55.3, 56.7, 66.5, 95.4, 110.4, 112.7, 114.0, 115.2, 116.3, 117.7, 118.2, 119.5, 120.4, 121.5, 122.7, 123.3, 124.2, 125.3, 127.2, 128.9, 129.3, 129.7, 130.2, 130.9, 132.4, 133.2, 134.2, 137.4, 138.2, 138.7, 139.4, 140.4, 163.9, 192.2, and 196.3. MS, m/z (%): 641 (M^+^, 10), 147 (48), and 31 (100). Anal. Calcd for C_40_H_27_N_5_O_4_ (641.69): C, 74.87; H, 4.24; and N, 10.91; found: C, C, 74.96; H, 4.34; and N, 11.02%.

### Evaluation of antioxidant property *via* DPPH

As mentioned above, in this research, the antioxidant property of some synthesized spiro-1,2,4-triazine such as **7a–7d** was investigated using DPPH free radical utilizing Shimada et al. (1992)’s procedures. According to the Shimada method, the concentration of spiro-1,2,4-triazine **7a–7d** was selected between 200 and 1,000 ppm, and a methanolic solution of DPPH (1 mmol/L) in equivalent volume was added to the spiro-1,2,4-triazine solution. The new mixture was mixed at room temperature, followed by placing it in a dark room after 30 min, where the absorbance of the mixture reached 517 nm. We compared the antioxidant activity of synthesized spiro-1,2,4-triazine **7a–7d** butylated hydroxytoluene (BHT) and 2-tertbutylhydroquinone (TBHQ) and instead of synthesized compounds, methanol (3 ml) was used. To measure the percentage of inhibition of the DPPH radical trapping experiment, the equation in the work of Yen and Duh (1994) was used.

### Evaluating the FRAP process of spiro-1,2,4-triazine antioxidant activity

Another way to look at the antioxidant properties of spiro-1,2,4-triazine is using the FRAP process that measures the amounts of iron (III) reduction by synthesized spiro-1,2,4-triazine **7a–7d** employing Yildirim et al.’s (2001) procedure. In this experiment, the spiro-1,2,4-triazine solution (1 ml), potassium ferricyanide (2.6 ml), and phosphate buffer (2.6 ml) were used to evaluate antioxidant activity according to Yildirim et al. (2001)’s procedure. The temperature of the mixture was maintained at 55°C for 35 min, followed by adding trichloroacetic acid (2.5 ml) to the new mixture and stirring it for 10 min. Finally, the absorbance of FeCl_3_ (0.6 ml) and the supernatant (2.5 ml) mixture in aqueous media (2.6 ml) as a sample was measured at 700 nm. The results showed that compounds with a high reducing ability have a greater power of absorbance. To confirm the calculations, they were computed three times. We ran the SPSS software version 18.0 to compute the analysis of variance (ANOVA) for synthesizing spiro-1,2,4-triazine data, which approved samples and standard variation. We also applied Duncan multiple-range experiments for separation with 95% (*p* < 0.05).

### Examining the antibacterial activity of the prepared spiro-1,2,4-triazine

To study the antibacterial activity of the prepared spiro-1,2,4-triazine, we prepared a Persian-type culture collection (PTCC) of Gram-positive and Gram-negative bacteria in Tehran, Iran, and for this reason, the disk diffusion procedure was utilized. For evaluating the antimicrobial ability of spiro-1,2,4-triazine, the two types of bacteria concentrations were similarly prepared according to the McFarland Standard No. 0.5 and were cultured for 16–24 h at 37°C. We used two standard drugs, namely, streptomycin and gentamicin, that killed bacteria. We prepared the suspension of bacteria with a sterile swab cultured on Mueller Hinton agar consistent with the McFarland Standard No. 0.5 (1.5 × 108 CFU/ml). Then, to deal with antibacterial properties, spiro-1,2,4-triazine (25 μg/ml) was added on sterile blank disks and the ready sample was placed for 24 h at 37°C in an incubator. We measured the diameter of inhibition and compared it with the standard sample.

### Ag/Fe_3_O_4_/CdO@MWCNT MNC application in the reduction of 4-NP

For this purpose, the mixture of Ag/Fe_3_O_4_/CdO@MWCNT MNCs (0.005 g) and 4-nitrophenol solution (25 ml, 2.5 mM) was stirred for 2 min at room temperature in the beaker and the newly produced NaBH_4_ (25 ml, 0.25 M) was added to the previous mixture as reducing agent, which could remove pollutants in the presence of the catalyst. After adding the aqueous NaBH_4_ to the first mixture, the solution color varied from pale yellow to lemon-colored. The stirring of the mixture was continued until the mixture became colorless. Next, for measuring the UV-Vis absorption, 1 ml of the solution was diluted to 25 ml at sure times. The concentration of 4-nitrophenol varied between 200 and 700 nm at room temperature and it was checked by the UV-Vis absorption spectra. The main point in the catalyst is its reusability in the same reactions. To confirm this point, the catalyst was removed from the mixture of reaction and washed with ethanol and finally dried to be reused in the same reaction.

## Results and discussion

In the current study, the new spiro-1,2,4-triazines **7** were produced with high efficiency by applying six component reactions of activated carbonyl compound **1**, methyl ketones **2**, diethyl oxalate **3**, ammonium acetate **4**, malononitrile **5**, and hydrazoyl chlorides **6** in aqueous media at room temperature in the vicinity of Ag/Fe_3_O_4_/CdO@MWCNT MNCs as a new reusable organometallic nanocatalyst. The catalytic activities of Ag/Fe_3_O_4_/CdO@MWCNT MNCs MNCs were evaluated *via* the synthesis of spiro-1,2,4-triazine derivatives in the presence of Ag/Fe_3_O_4_/CdO@MWCNT MNCs. The important issue in all organic reactions lies in achieving the best condition for conducting the reactions. To achieve this purpose, we initially chose the multicomponent reaction of 4-methoxyacethophenone **1a**, diethyl oxalate **2**, ammonium acetate **3**, ninhydrin **4**, malononitrile **5**, and hydrazoyl chloride **6a** as a model reaction (Table 1). Without the catalyst even after 10 h, the production of compound **7a** was not carried out (entry 1, Table 1). To optimize the temperature of the sample reaction and achieve the best temperature, the reaction temperature was increased to 100°C but it did not exhibit significant variation in the efficiency of spiro-1,2,4-triazine **7a** (Entry 2, Table 1). Also, such reactions did not take place without the catalyst. To confirm this point, CdO-NPs (0.01 g) as catalysts were added to the mixture of reaction. After 4 h, spiro-1,2,4-triazine **7a** was generated with good efficiency (entry 4, Table 1). Therefore, these reactions needed a catalyst for their performance. To find out the best catalyst for the model reaction, we considered many nanocatalysts, including Ag NPs, Fe_3_O_4_ MNPs, CdO NPs, Fe_3_O_4_/CdO NPs, Fe_3_O_4_/CdO/MWCNTs, Ag@MWCNTs, MWCNTs, Ag NPs, and Ag/Fe_3_O_4_/CdO@MWCNTs. Among these catalysts, Ag/Fe_3_O_4_/CdO@MWCNTs was selected as the nanocatalyst for the synthesis of spiro-1,2,4-triazine **7a**, and the production efficiency was increased by this catalyst. Ag/Fe_3_O_4_/CdO@MWCNTs as a catalyst have two sites in their structure. The three sites in the synthesized catalyst (Ag, Fe, and Cd) are Lewis acids and are caused by the activation of carbonyl groups. According to the results shown in Table 1, Ag NPs are more useful compared with Fe_3_O_4_, CdO, Fe_3_O_4_/CdO, and Fe_3_O_4_/CdO/MWCNT. Ag is a stronger Lewis acid than Fe_3_O_4_ and TiO_2_ and is very significant by which these reactions were conducted with catalytic amounts of Ag/Fe_3_O_4_/CdO/MWCNTs-MNCs. Therefore, increasing Ag/Fe_3_O_4_/CdO/MWCNTs-MNCs amounts from 0.02 to 0.03 g did not show any remarkable variation in the efficiency of the reaction. So, 0.02 g of Ag/Fe_3_O_4_/CdO/MWCNTs-MNCs was needed for the preparation of spiro-1,2,4-triazine with high efficiency (entry 11, Table 1) and the yield of compound **7a** is 95% after 3 h (entry 11, Table 1). The role of nanocatalysts in the preparation of spiro-1,2,4-triazine derivatives are Lewis acid and Lewis base. Ag, Cd, and Fe as Lewis acids activate the carbonyl group for nucleophilic attack. As displayed in Table 1, among Lewis acids, Ag is more effective compared with Cd and Fe.

**TABLE 1:** Determining the most optimal conditions, including catalyst, amount of catalyst, and temperature for the synthesis of **7a**.

Entry	Catalyst	Temp. (°C)	Catalyst (g)	Time (h)	Yield %^a^
1	None	r.t.	—	10	—
2	None	100	—	8	—
3	CdO-NPs	r.t.	0.01	4	45
4	CdO-NPs	r.t.	0.015	4	58
5	CdO-NPs	r.t.	0.02	4	65
6	Fe_3_O_4_-MNPs	r.t.	0.015	4	35
7	Ag NPs	r.t.	0.015	4	78
8	MWCNT	r.t.	0.015	4	27
9	CuO-NPs	r.t.	0.015	4	38
10	Ag/Fe_3_O_4_/CdO	r.t.	0.015	4	80
11	Fe_3_O_4_/CdO	r.t.	0.015	4	70
12	Ag/CdO@ MWCNT	r.t.	0.015	4	78
13	Fe_3_O_4_/CdO/MWCNT	r.t.	0.015	4	70
14	CdO@ MWCNT	r.t.	0.015	4	68
15	Ag/Fe_3_O_4_/MWCNT	r.t.	0.015	4	75
16	Ag/Fe_3_O_4_/CdO@MWCNT	r.t.	0.015	4	87
17	Ag/Fe_3_O_4_/CdO@MWCNT	r.t.	0.02	4	95
18	Ag/Fe_3_O_4_/CdO@MWCNT	r.t.	0.025	4	95
19	Ag/Fe_3_O_4_/CdO@MWCNT/Et3N	r.t.	0.02	3	95

^a^ isolated yields.

Because of the easy and simple extraction of Fe_3_O_4_ magnetic nanoparticles (MNPs) from the mixture of reactions, their application in several reactions is very significant. Additionally, in this inquiry, the solvent’s effects on the synthesis of compound **7a** in the presence of Ag/Fe_3_O_4_/CdO@MWCNT (0.02 g) were investigated. The results in Table 2 display that water is the best solvent to carry out the reaction.

**TABLE 2:** Determining the best solvent for the generation of **7a**.

Entry	Solvent	Time (h)	Yield %^a^
1	EtOH	15	None
2	CH_2_Cl_2_	8	60
3	CHCl_3_	5	68
4	H_2_O	3	95
5	Solvent free	8	58
6	DMF	12	30
7	Toluene	12	68
8	CH_3_CN	5	90

^a^ isolated yields.

As illustrated in Tables 1 and 2, Ag/Fe_3_O_4_/CdO@MWCNT-MNC (0.02 g) as an organometallic catalyst, room temperature, and aqueous media are the suitable conditions for the generation of spiro-1,2,4-triazine **7**. Reusing the synthesized catalysts is an important factor in the synthesis of organic compounds. In this research, the synthesized nanocatalyst was reused five times for the synthesis of spiro-1,2,4-triazine **7a** (Table 3). The final results attested that the catalyst could be used again five times with negligible change in its power (Table 3). To use the magnetic nanocatalyst again, we require the exterior magnet to separate the catalyst from the mixture of reactions, followed by washing the catalyst with water and drying it at room temperature for 24 h, and using it another time.

**TABLE 3:** How often the catalyst is reused for the synthesis of compound **7a**.

Run	% yield^a^
First	95
Second	95
Third	92
Fourth	90
Fifth	87

^a^ isolated yields.

**SCHEME 2:**
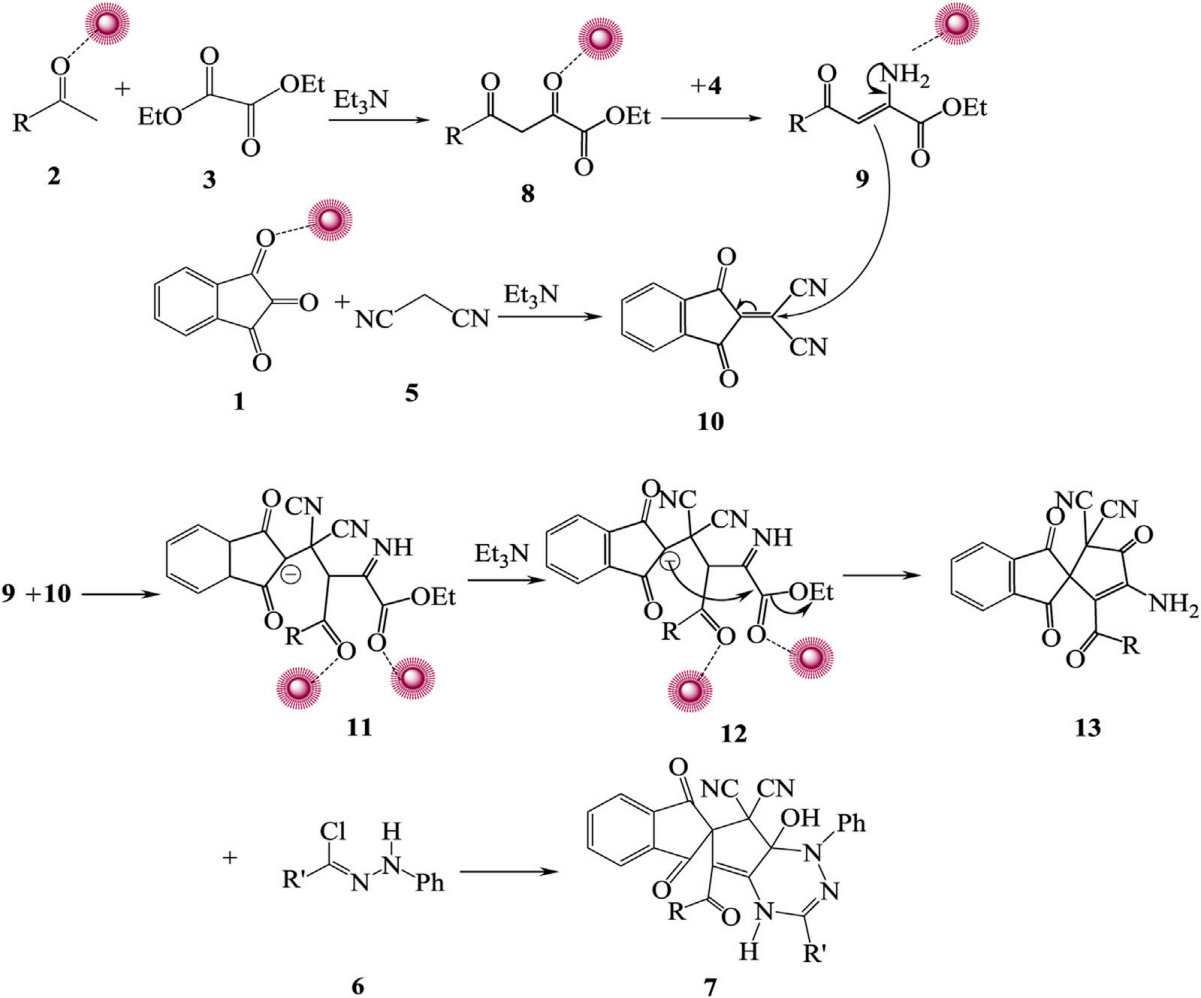
Recommended mechanism for preparation of **7**. First, acetophenones **2** and diethyl oxalate **3** reacted at room temperature accompanied by Ag/Fe_3_O_4_/CdO@MWCNT MNCs, and 1,3-dicarbonyl **8** was generated. Ammonium acetate **4** reacted with the carbonyl group of intermediate **8** and produced iminium ion **9** that reacted with additional intermediate **10** from the reaction of carbonyl compound **1** reacting with malononitrile 5 and generating intermediate **11**. Intermolecular cyclization of intermediate **12** made intermediate **13** that reacted with hydrazoyl chloride **6** produced spiro-1,2,4-triazine **7**.

After each run, to synthesize compound **7a**, the catalyst was removed from the mixture of reactions, washed, and used again. For this reason, the yield of compound **7a** decreased after five times because of the reduced amount of catalyst and its separation after each run. It should be mentioned that after the separation of the catalyst, the amount of the catalyst might be changed but not its form and size. Lowering the ratios of the catalyst has the most impact on the efficiency of compound **7a**. For confirmation, the structure of synthesized spiro-1,2,4-triazine **7**, ^1^H NMR, ^13^C NMR, and IR, elemental analysis and mass spectrum were employed. At 3.78, 3.83, and 3.87 ppm in the ^1^H NMR spectra of spiro-1,2,4-triazine **7a** displayed three singlets for methoxy protons. The one singlet appeared at 10.32 ppm for NH proton and several signals for aromatic protons at 6.98–8.22 ppm. The carbonyl moiety displayed four resonances in the ^13^C NMR spectra of **7a** at 164.2, 165.2, 167.3, and 187.2 ppm. Also, another route for confirming the existence of carbonyl groups in the construction of synthesized compounds is the IR spectrum. The preparation mechanism for synthesized compounds 7 is recommended as in Scheme 2.

### Evaluating the antioxidant property of prepared spiro-1,2,4-triazine by DPPH

**FIGURE 1:**
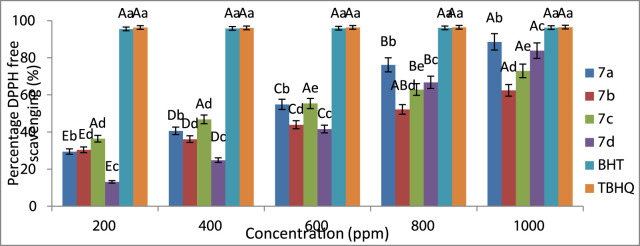
Order of the antioxidant activity of **7a–7d** using DPPH.

Furthermore, this investigation aimed at examining the antioxidant property of synthesized spiro-1,2,4-triazines, and DPPH was used to realize this goal. It should be mentioned that we applied DPPH radical scavenging test for many purposes such as the antioxidant activity of synthesized organic compounds, foods, and biological structures (Bidchol et al., 2011; Saundane and Nandibeoor, 2015) by taking electron or hydrogen atoms by free radical of DPPH. The synthesized spiro-1,2,4-triazine loses the hydrogen atom or one electron in the presence of the DPPH radical, meaning that these compounds have antioxidant properties. The percentage of trapping the DPPH free radical by the synthesized spiro-1,2,4-triazine displays the order of antioxidant activity. Through this inquiry, we examined the antioxidant activity of some synthesized compounds such as **7a–7d** and compared them with standard synthesized antioxidant BHT and TBHQ, where the electron or hydrogen absorbance of these compounds *via* DPPH free radical proved their antioxidant activity. If an electron or hydrogen atom is adsorbed *via* DPPH, its absorbance decreases by 517 nm. Overall, the antioxidant ability of spiro-1,2,4-triazine derivatives **7a–7d** obtained was as TBHQ ≈ BHT>**7a** > **7d** > **7c** > **7b** (Figure 1).

Figure 1 showed that good differences existed between the spiro-1,2,4-triazine concentration of BHT and TBHQ as standard antioxidants. Compound **7a** showed a good ability relative to BHT and TBHQ among experimented spiro-1,2,4-triazine **7a–7d**.

### Assessment of spiro-1,2,4-triazine antioxidant activity using Fe^3+^ reduction

**FIGURE 2:**
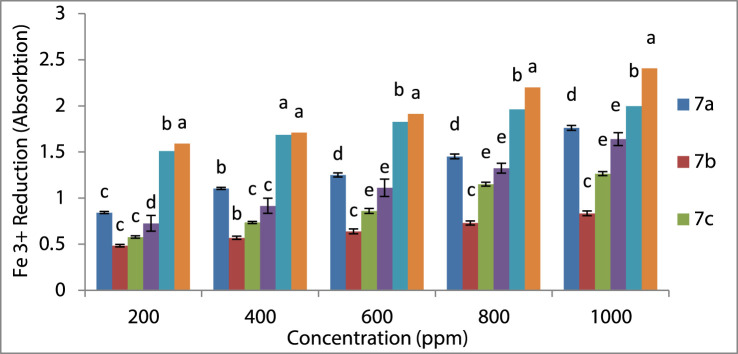
Ferric ion (Fe^3+^) decreasing antioxidant ability (FRAP) of compounds **7a–7d**.

The antioxidant property of spiro-1,2,4-triazine **7a–7d** was tested by another procedure for confirming it. Spiro-1,2,4-triazine caused the reduction of ferric ions (Fe^3+^) and amounts of reduction were measured based on the reduction of Fe^3+^/ferricyanide to the Fe^2+^/ferrous at 700 nm^[51]^ and spiro-1,2,4-triazine **7a** exhibited good effect compared with BHT and TBHQ. Figure 2 shows the order of spiro-1,2,4-triazine **7a–7d** antioxidant activity as TBHQ > BHT > **7a** > **7d** > **7c** > **7b**.

### Antibacterial activity evaluation of synthesized spiro-1,2,4-triazine

To study the antibacterial activity of synthesized compounds, we used two antibiotic drugs, namely, streptomycin and gentamicin and compared the results of the antimicrobial synthesized compounds with two standards as displayed in Table 4. For the evaluation of this experiment, two suitable and significant factors that affect the diameter and inhibition zone are the type of bacteria and the concentration of synthesized spiro-1,2,4-triazines. Among the Gram-positive and negative bacteria, spiro-1,2,4-triazines **7b**, **7d**, **7f**, and **7g** affect *Escherichia coli* owing to a good diameter of the inhibition zone.

**TABLE 4:** Antibacterial activity of some synthesized compounds **7**.

Compound	*Staphylococcus aureus* (+)	*Bacillus cereus* (+)	*E. coli* (−)	*Klebsiella pneumoniae* (−)
**7a**	6	8	8	6
**7b**	17	19	22	17
**7c**	10	9	9	8
**7d**	18	21	22	16
**7e**	10	8	10	7
**7f**	18	22	21	18
**7g**	20	21	23	18
Streptomycin	21	23	22	22
Gentamicin	22	22	23	21

## Conclusion

This study approached effective, green, and environment-friendly reactions, namely, acetophenones, diethyl oxalate, ammonium acetate, ninhydrin, malononitrile, and hydrazoyl chlorides in aqueous media at an ambient temperature in the presence of the new organometallic nanocatalyst Ag/Fe_3_O_4_/CdO@MWCNT-MNC, which generated new derivatives of spiro-1,2,4-triazines. Also, two methods were employed for the evaluation of the antioxidant power of the synthesized spiro-1,2,4-triazines **7a–7d**. DPPH radical trapping and reducing Fe^+3^ by synthesized compounds, these two procedures confirmed that the synthesized compounds had good antioxidant abilities relative to standard antioxidants. Moreover, to examine the antibacterial activity of synthesized compounds, we utilized the Gram-positive and negative bacteria, and to confirm the antimicrobial ability of the produced spiro-1,2,4-triazines, we relied on the disk diffusion process. The results of the antioxidant and antimicrobial activity investigations display that synthesized spiro-1,2,4-triazines have good biological activity and could prevent bacterial growth. Therefore, this procedure that is used for the synthesis of spiro-1,2,4-triazines carries several advantages, including reactions with high rate, high yield output, green processes, using low amounts of catalyst, easy separation of organometallic catalyst from the mixture of reaction, and easy product purification, which are the important aspects in these reactions.

## Data Availability

The original contributions presented in the study are included in the article/Supplementary Material; further inquiries can be directed to the corresponding author.
