# Supplementary material for: Synthesis and evaluation of the antioxidant activity of new spiro-1,2,4-triazine derivatives applying Ag/Fe3O4/CdO@MWCNT MNCs as efficient organometallic nanocatalysts
Source: Front Chem. 2022 Sep 29;10:1001707. doi: 10.3389/fchem.2022.1001707 (PMC9574876; doi:10.3389/fchem.2022.1001707)
Supplement: Supplementary file 1 [file DataSheet1.docx]

Synthesis and Evaluation of Antioxidant Activity of New Spiro-1,2,4-Triazine Derivatives Applying Ag/Fe_3_O_4_/CdO@MWCNTs MNCs as Efficient Organometallic Nano-catalyst

Elham Ezzatzadeh^a*^, Somayeh Soleimani-Amiri^b^, Zinatossadat Hossaini^c^, Khatereh Khandan Barani^d^

*^a^Department of Chemistry, Ardabil Branch, Islamic Azad University, Ardabil, Iran*

*^b^Department of Chemistry, Karaj Branch, Islamic Azad University, Karaj, Iran*

*^c^Department of Chemistry, Qaemshahr Branch, Islamic Azad University, Qaemshahr, Iran*

*^d^Department of Chemistry, Zahedan Branch, Islamic Azad University, Zahedan, Iran*

**Corresponding author: E-mail addresses:* [*dr.ezzatzadeh@yahoo.com*](mailto:dr.ezzatzadeh@yahoo.com)


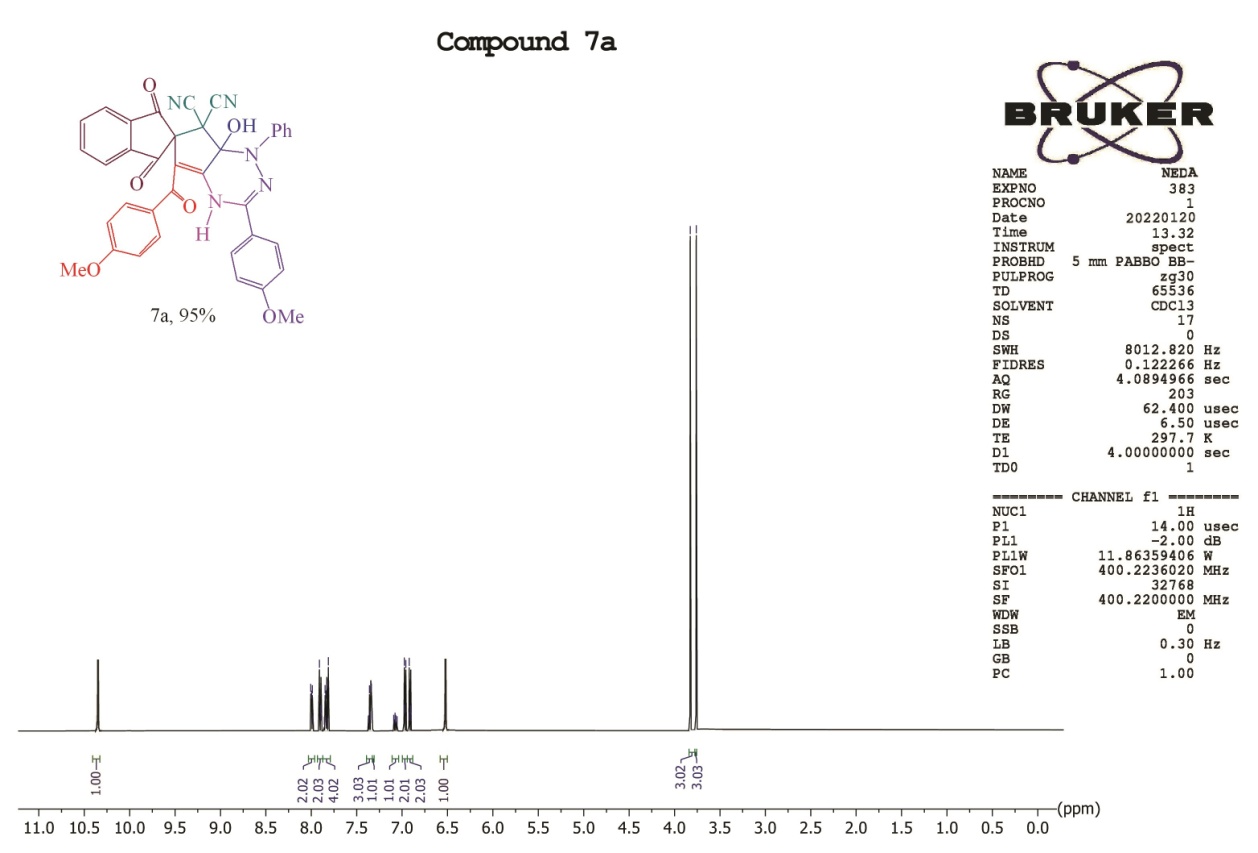


^1^HNMR Compound **7a**


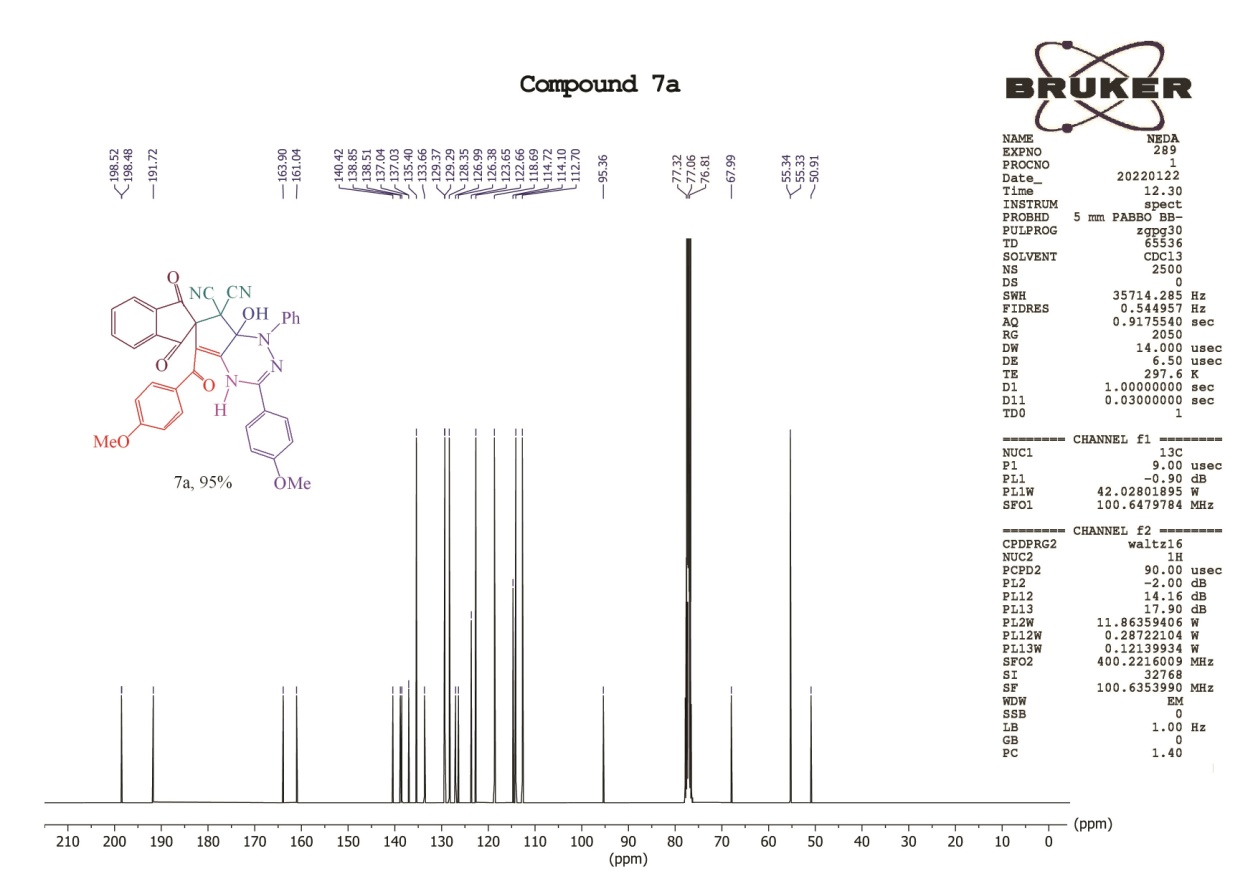


^13^CNMR Compound **7a**

^
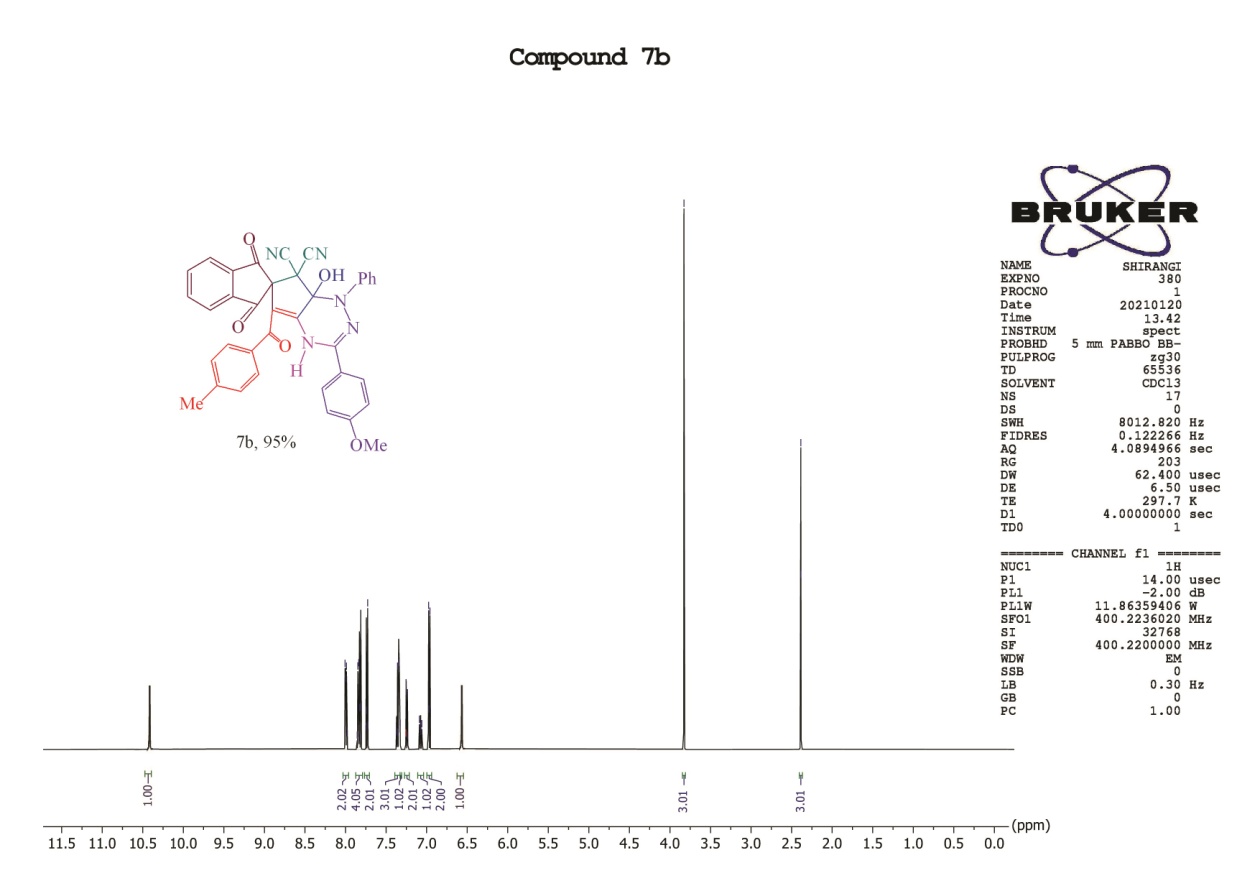
^

^1^HNMR Compound **7b**


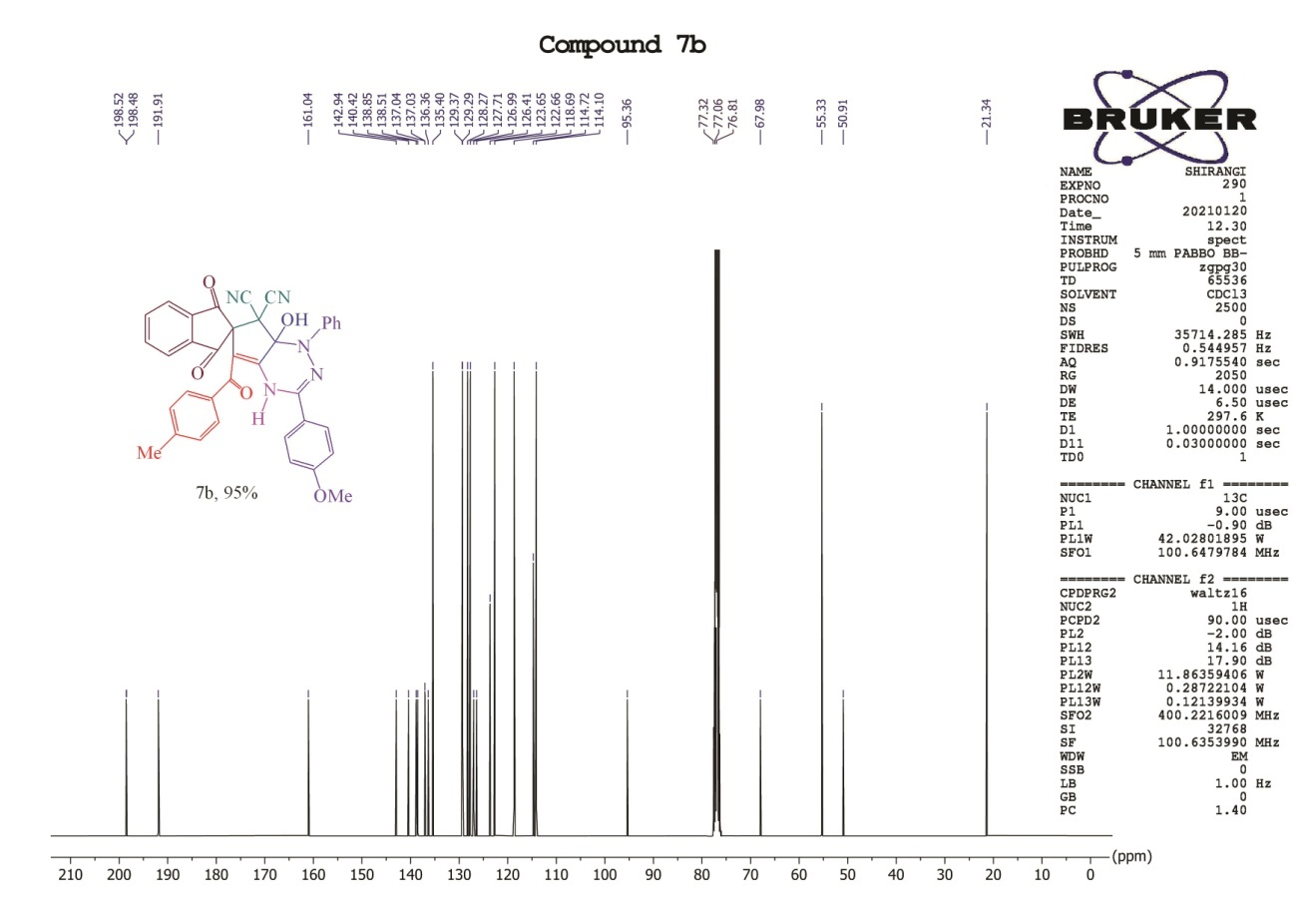


^13^CNMR Compound **7b**


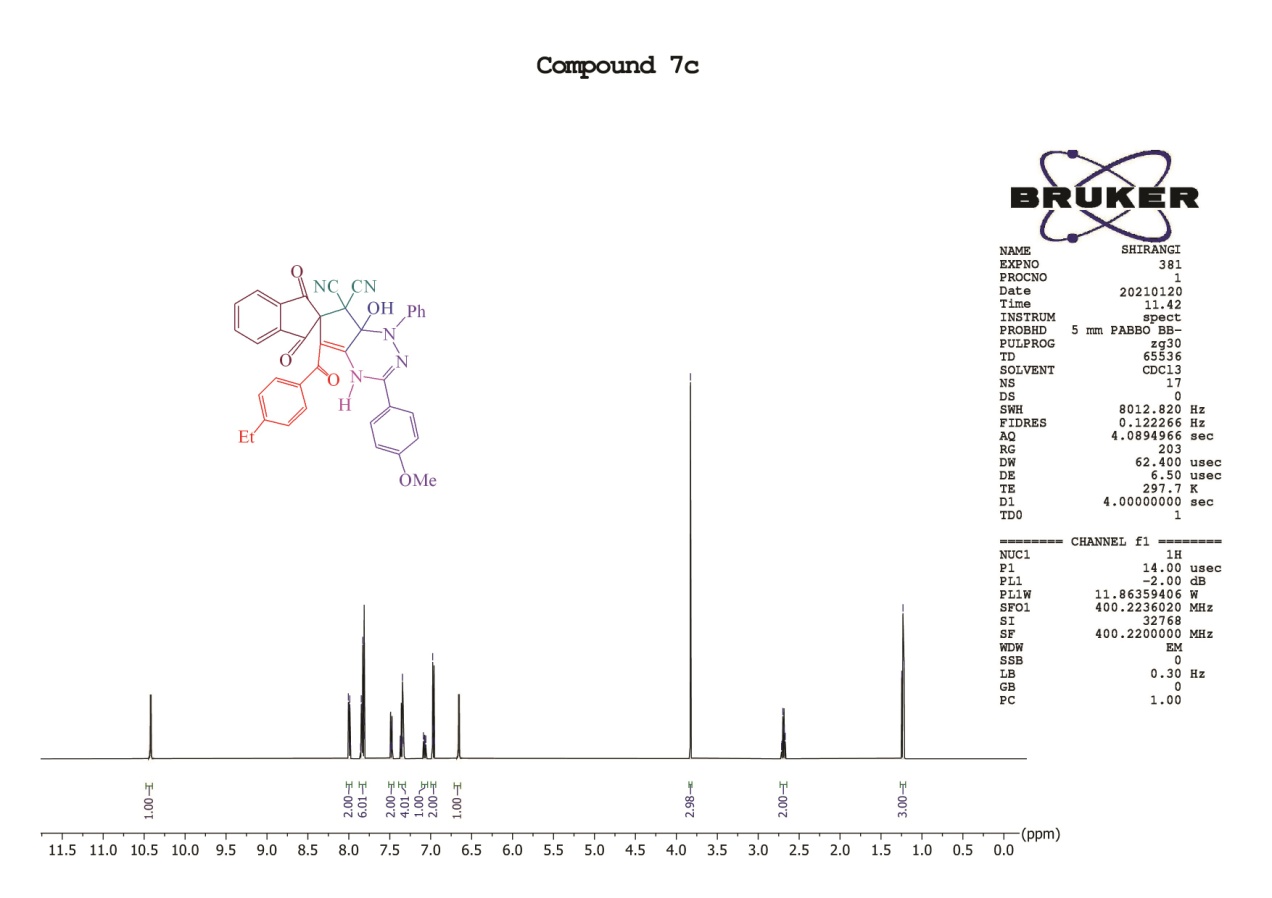


^1^HNMR Compound **7c**


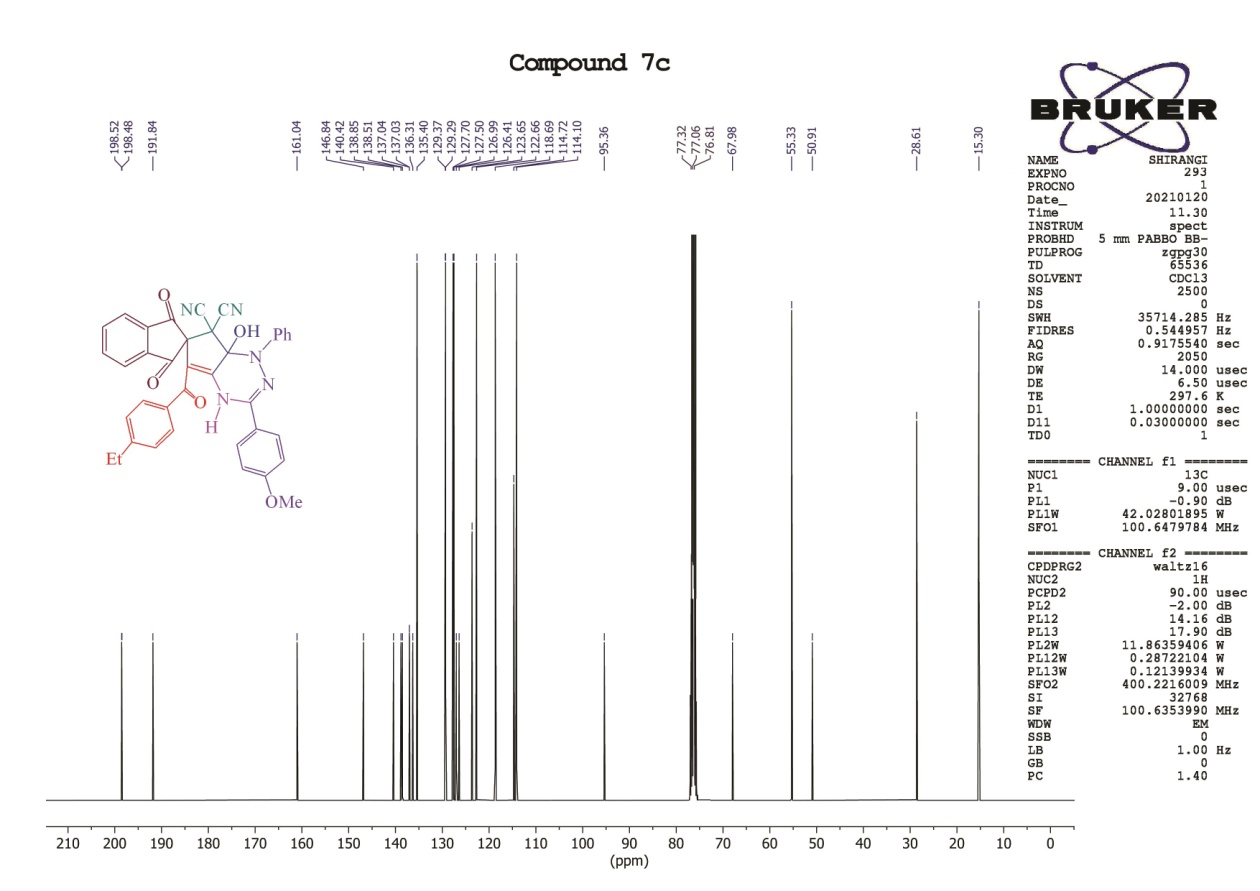


^13^CNMR Compound **7c**


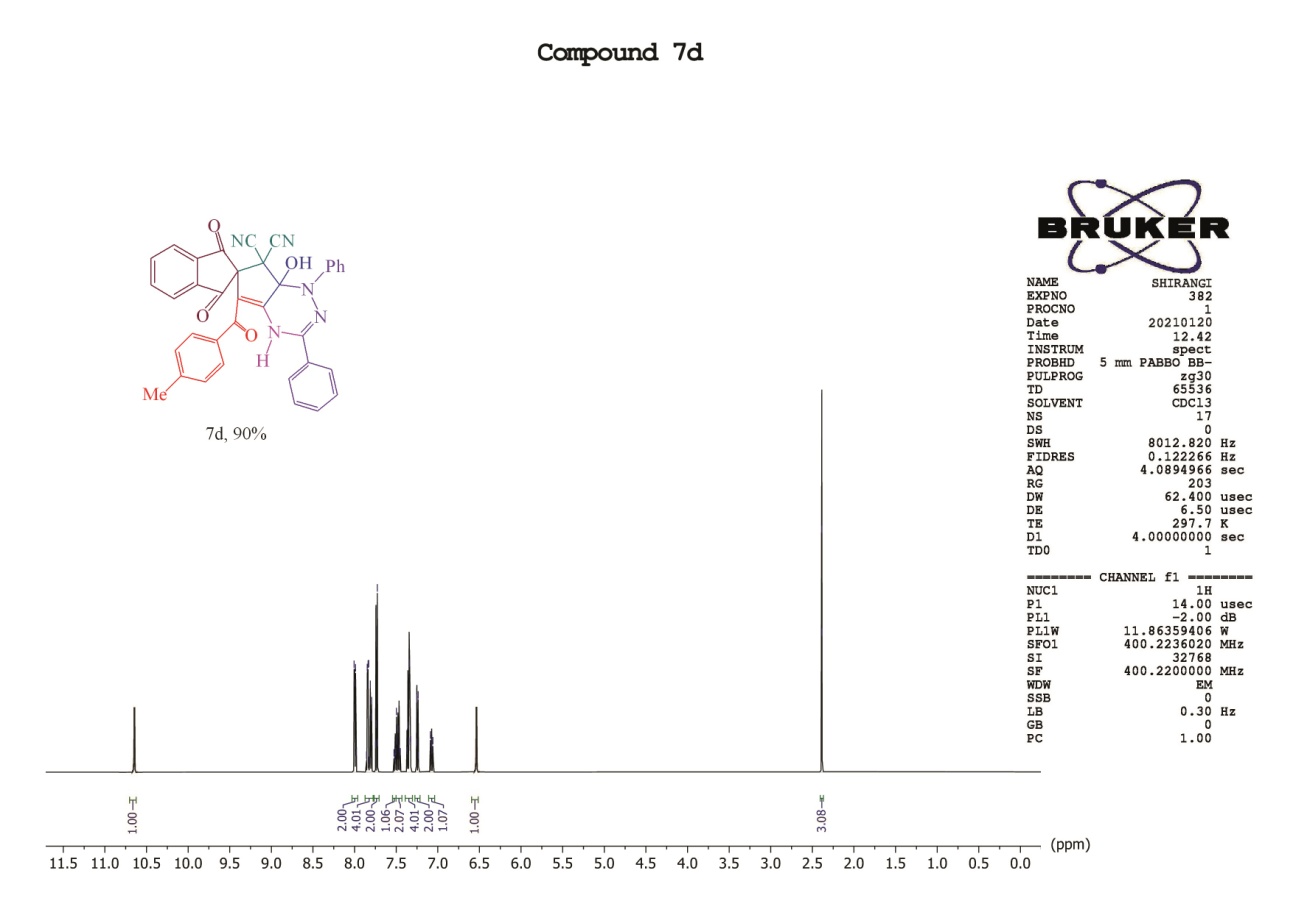


^1^HNMR Compound **7d**


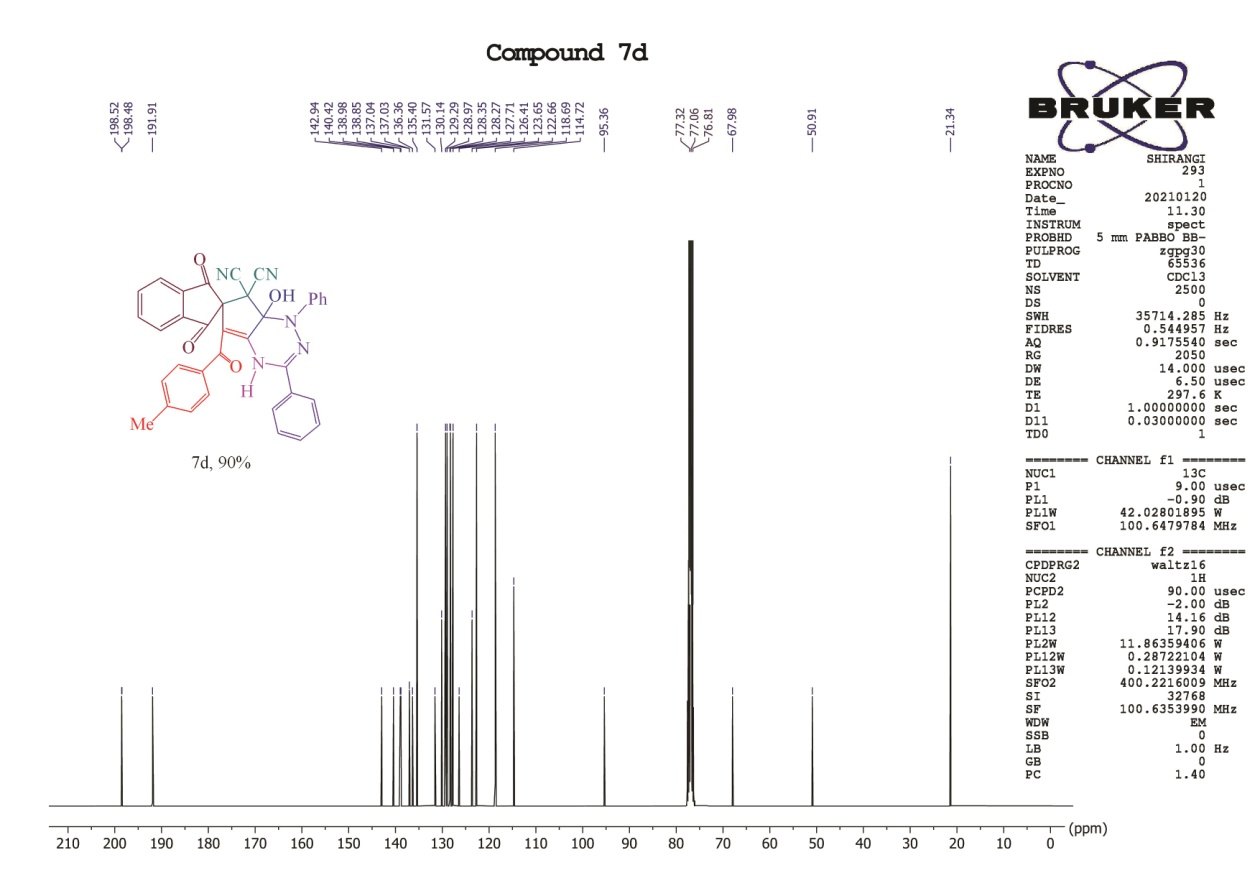


^13^CNMR Compound **7d**

^
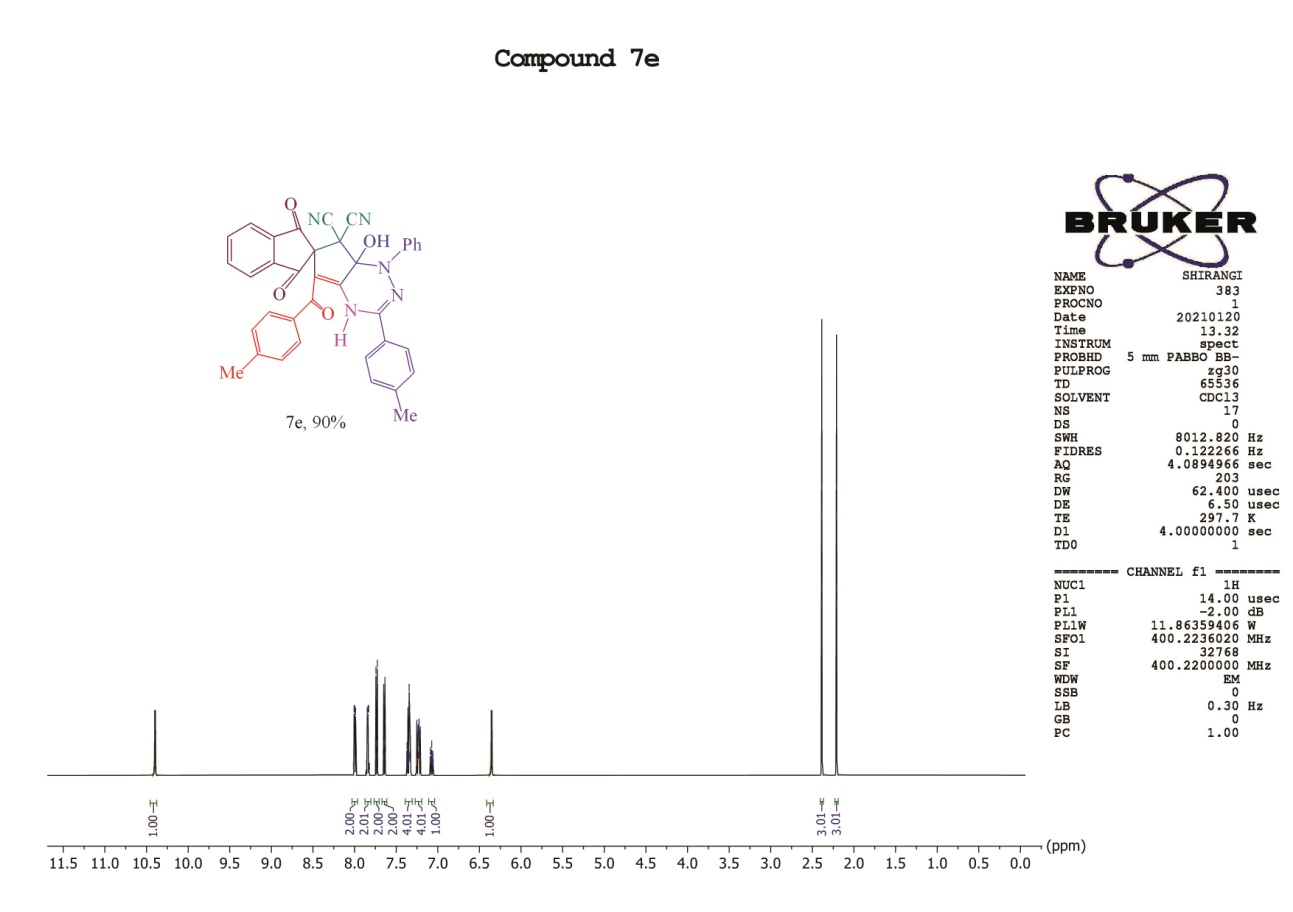
^

^1^HNMR Compound **7e**


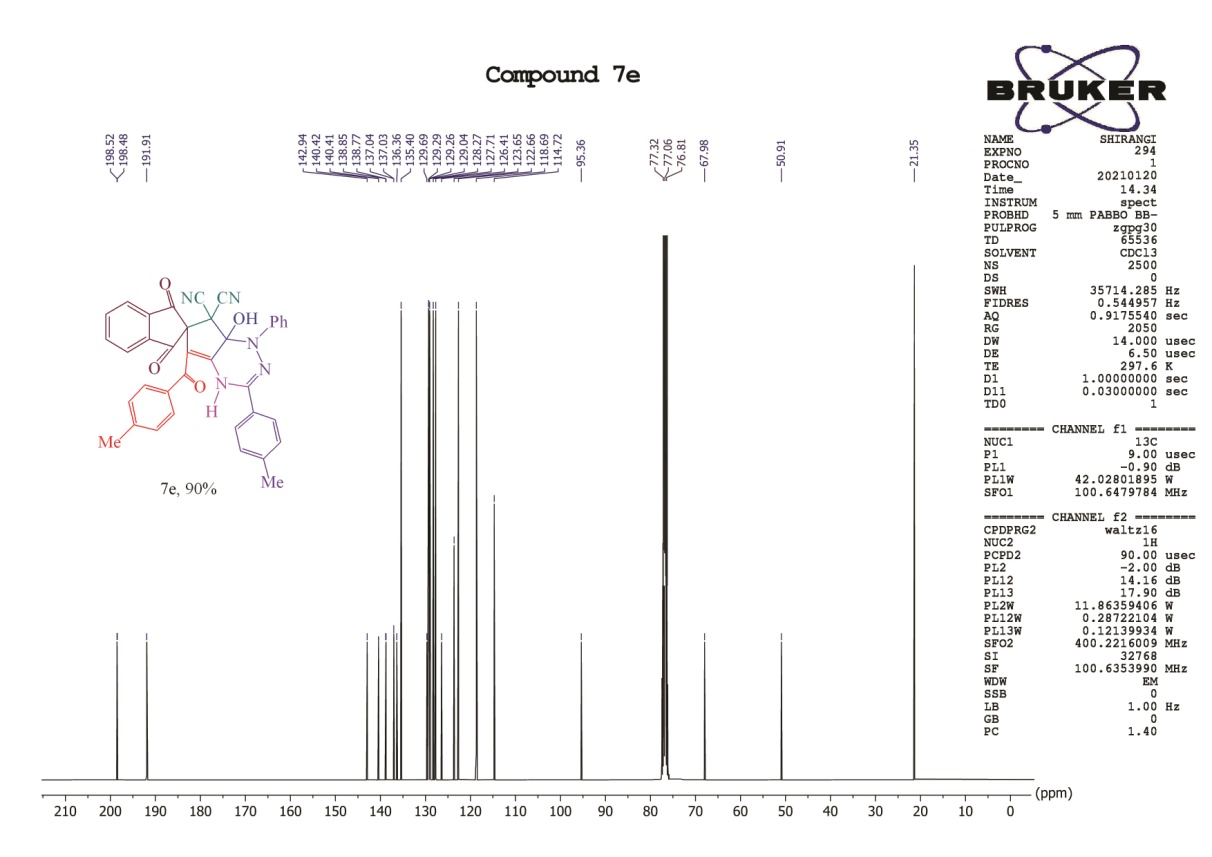


^13^CNMR Compound **7e**

^
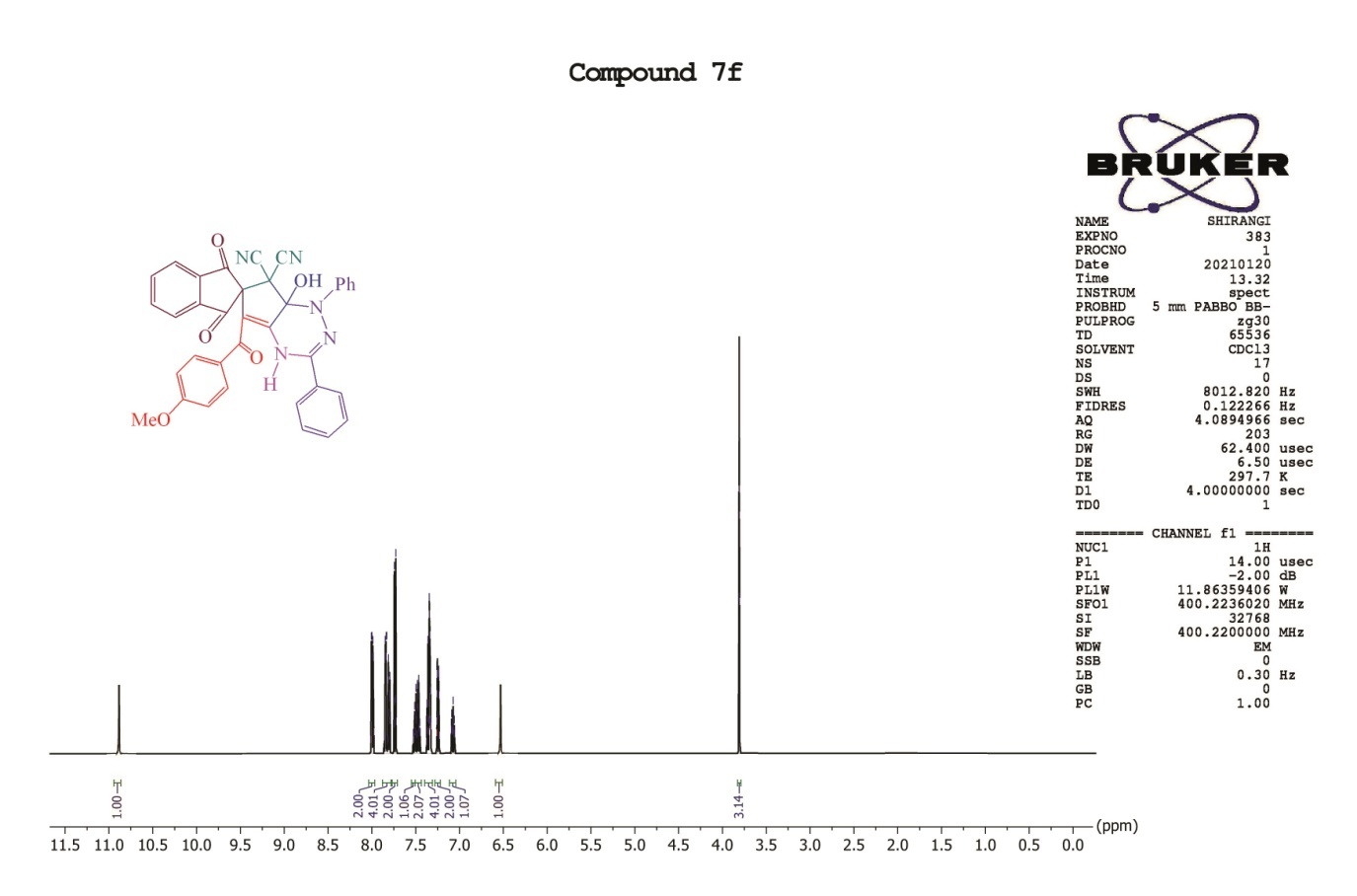
^

^1^HNMR Compound **7f**


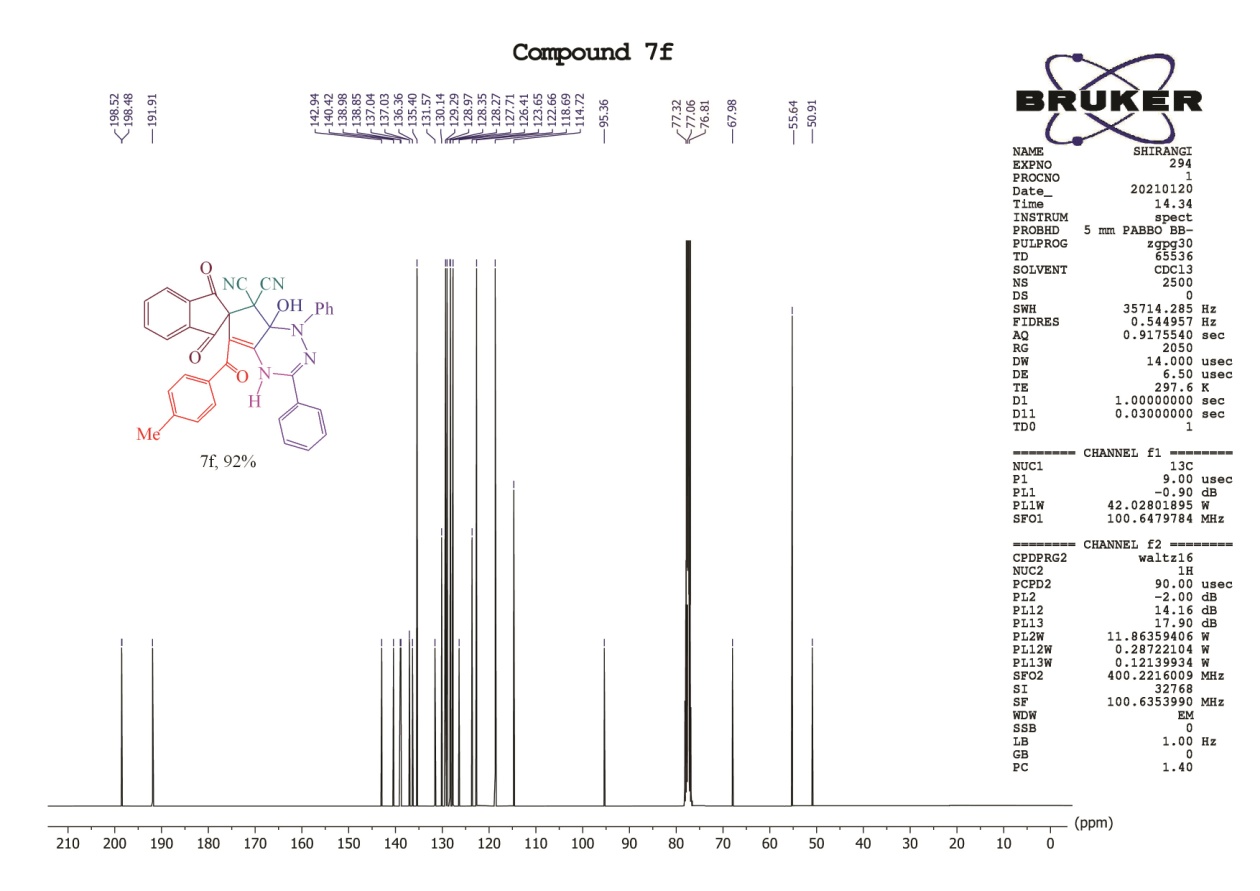


^13^CNMR Compound **7f**

^
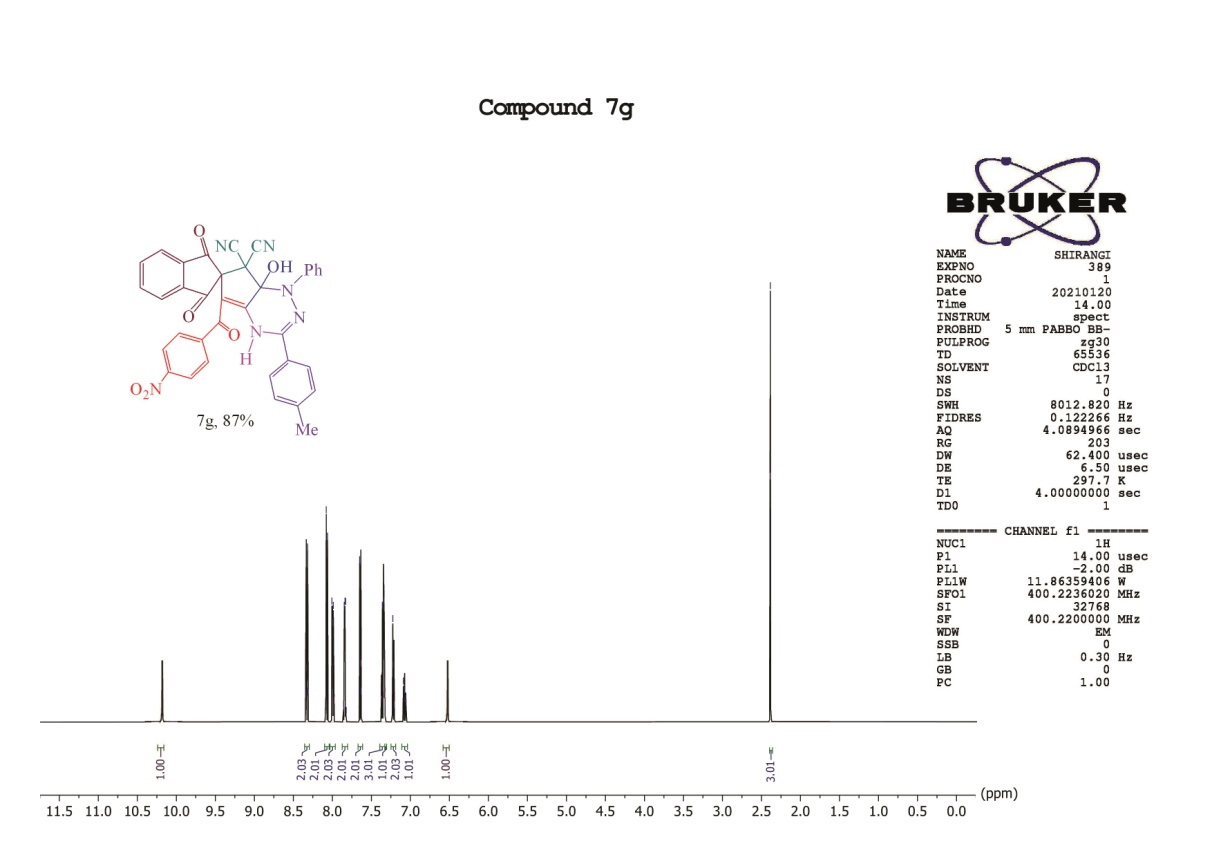
^

^1^HNMR Compound **7g**


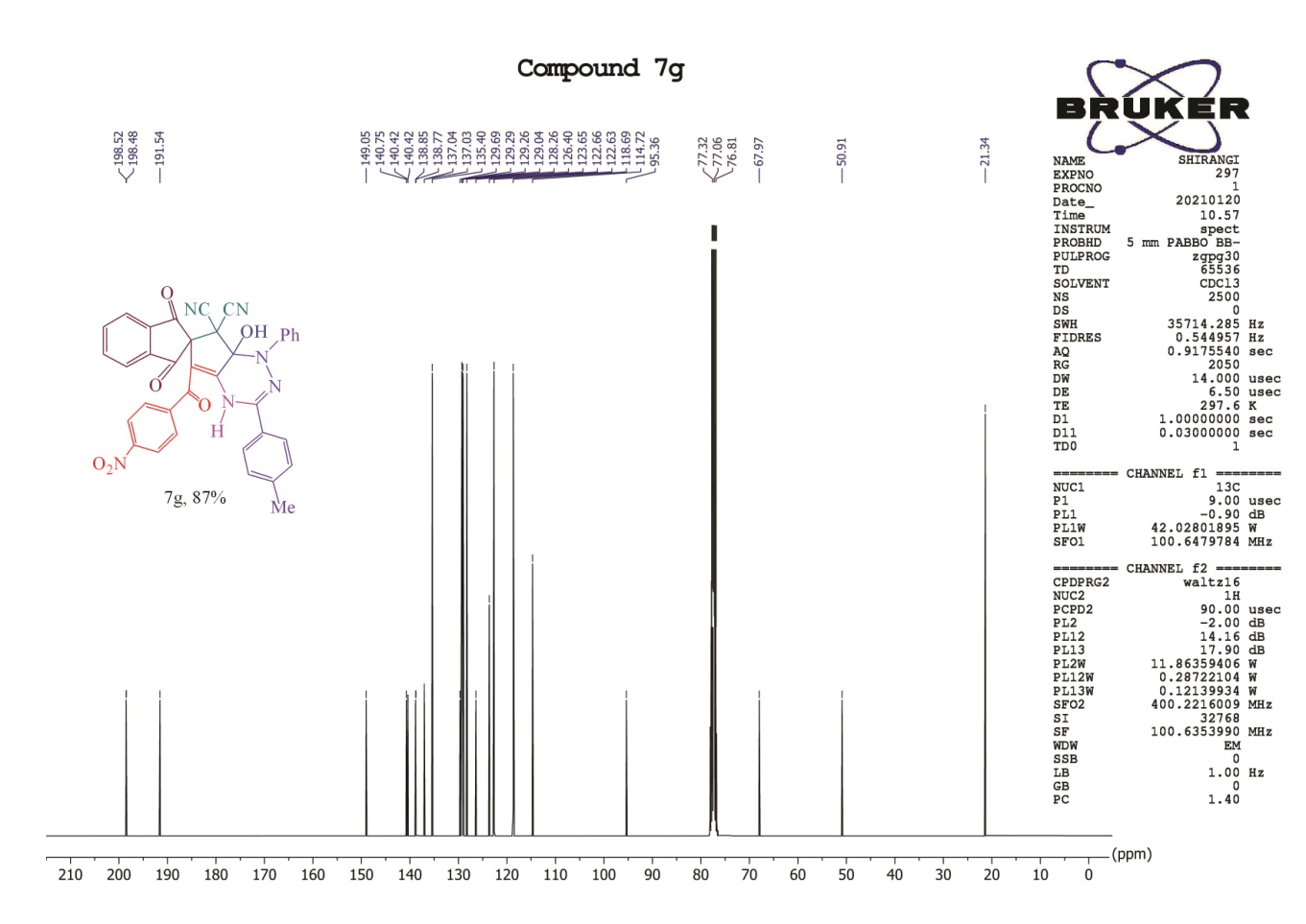


^13^CNMR Compound **7g**

^
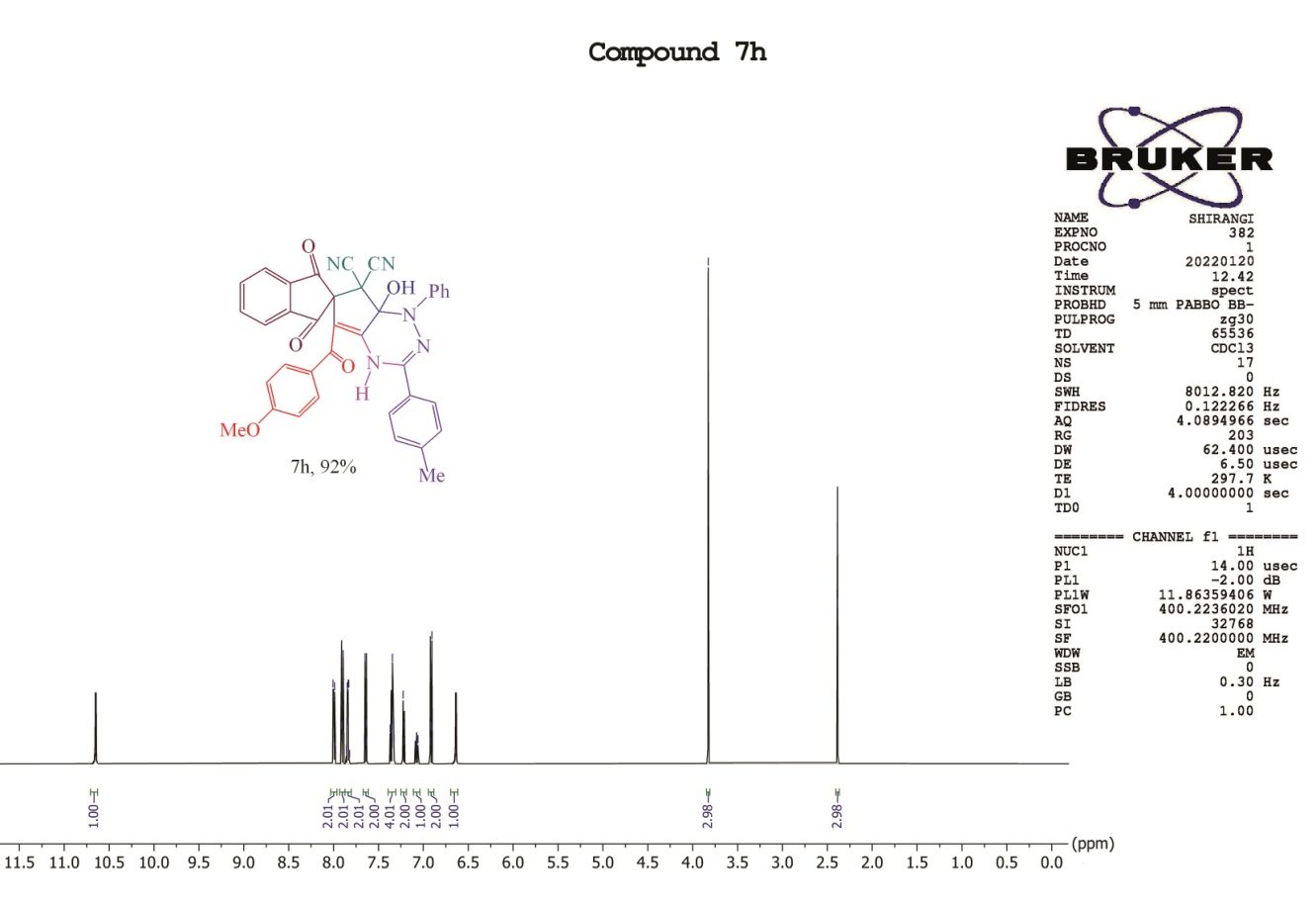
^

^1^HNMR Compound **7h**


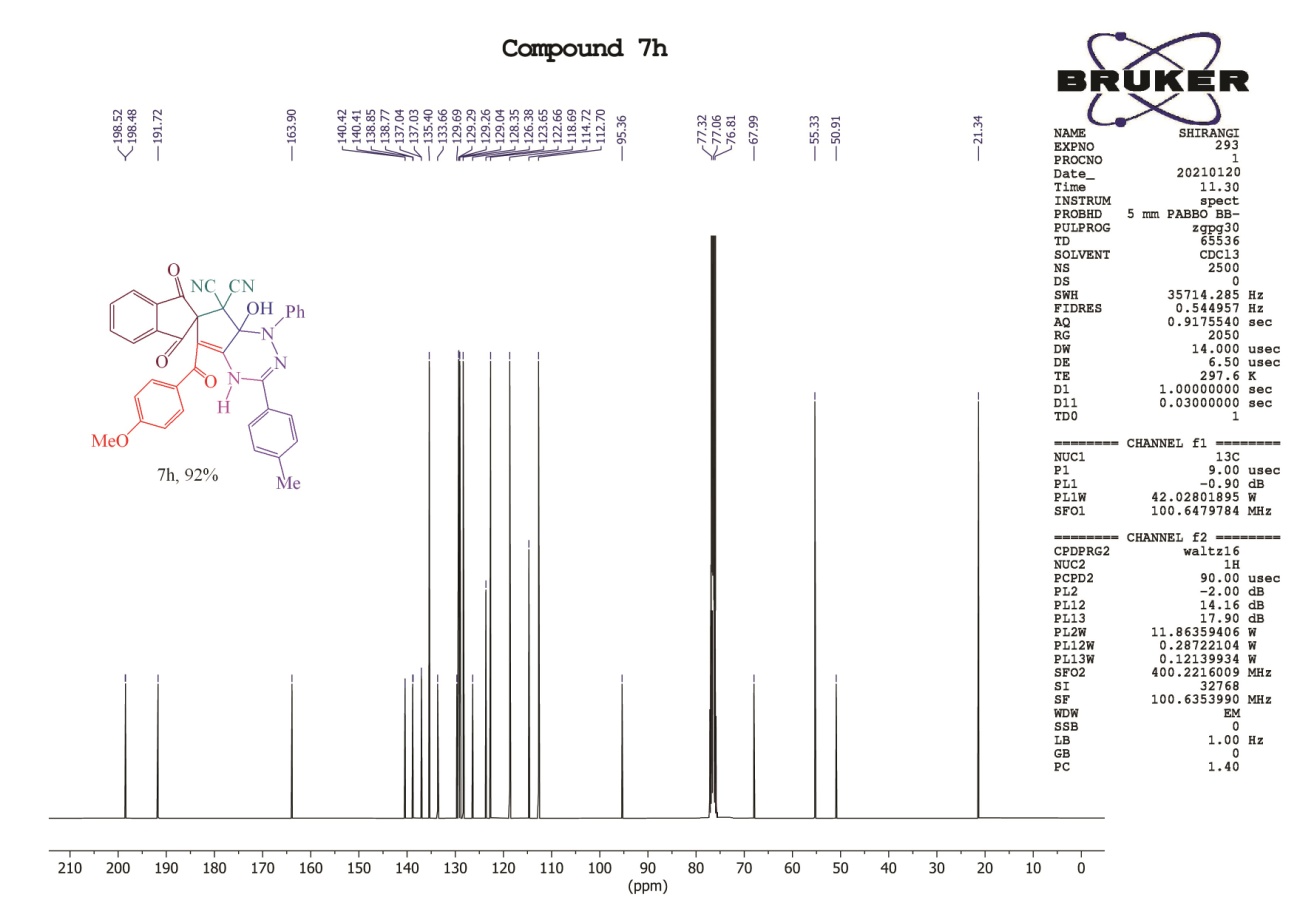


^13^CNMR Compound **7h**

^
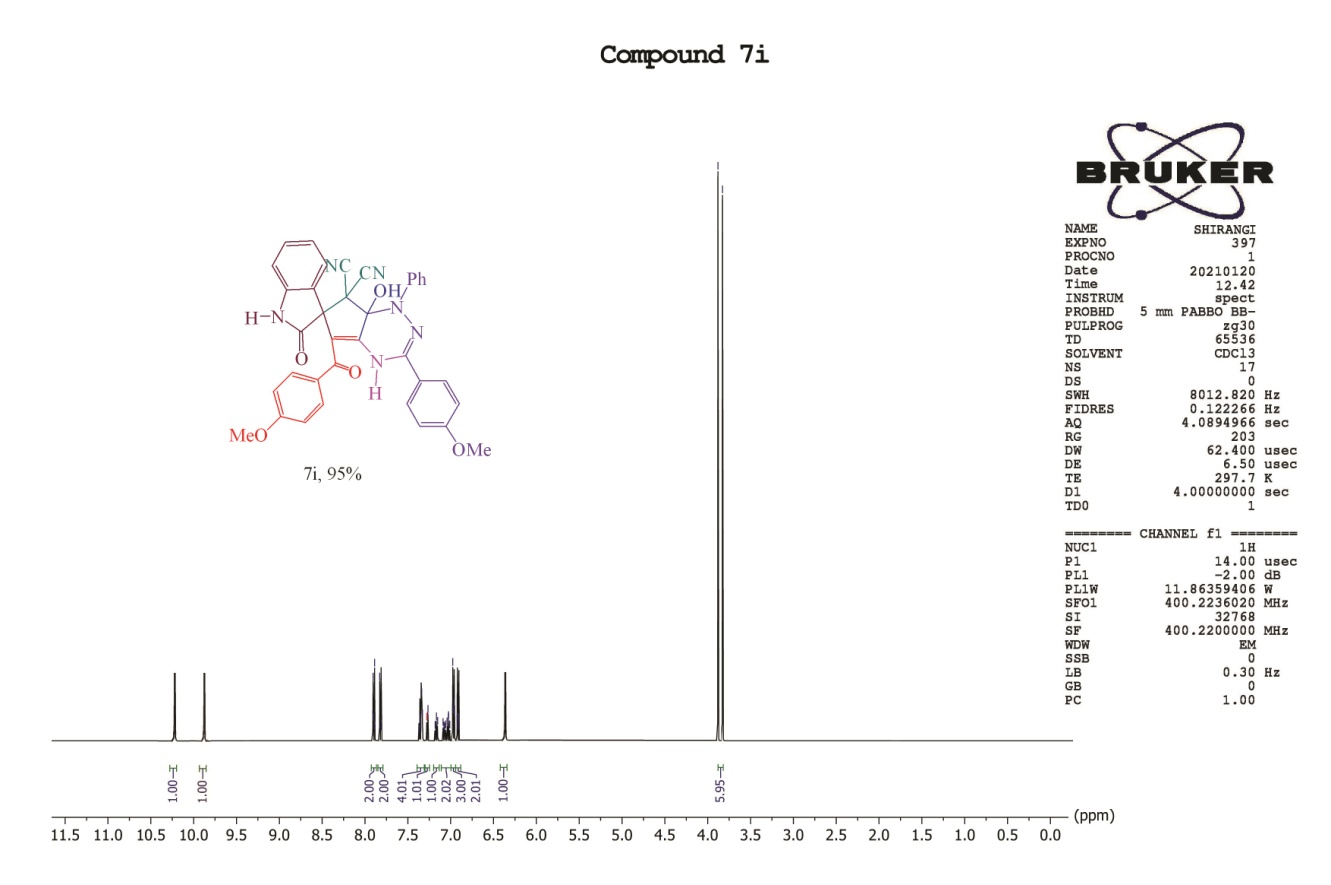
^

^1^HNMR Compound **7i**


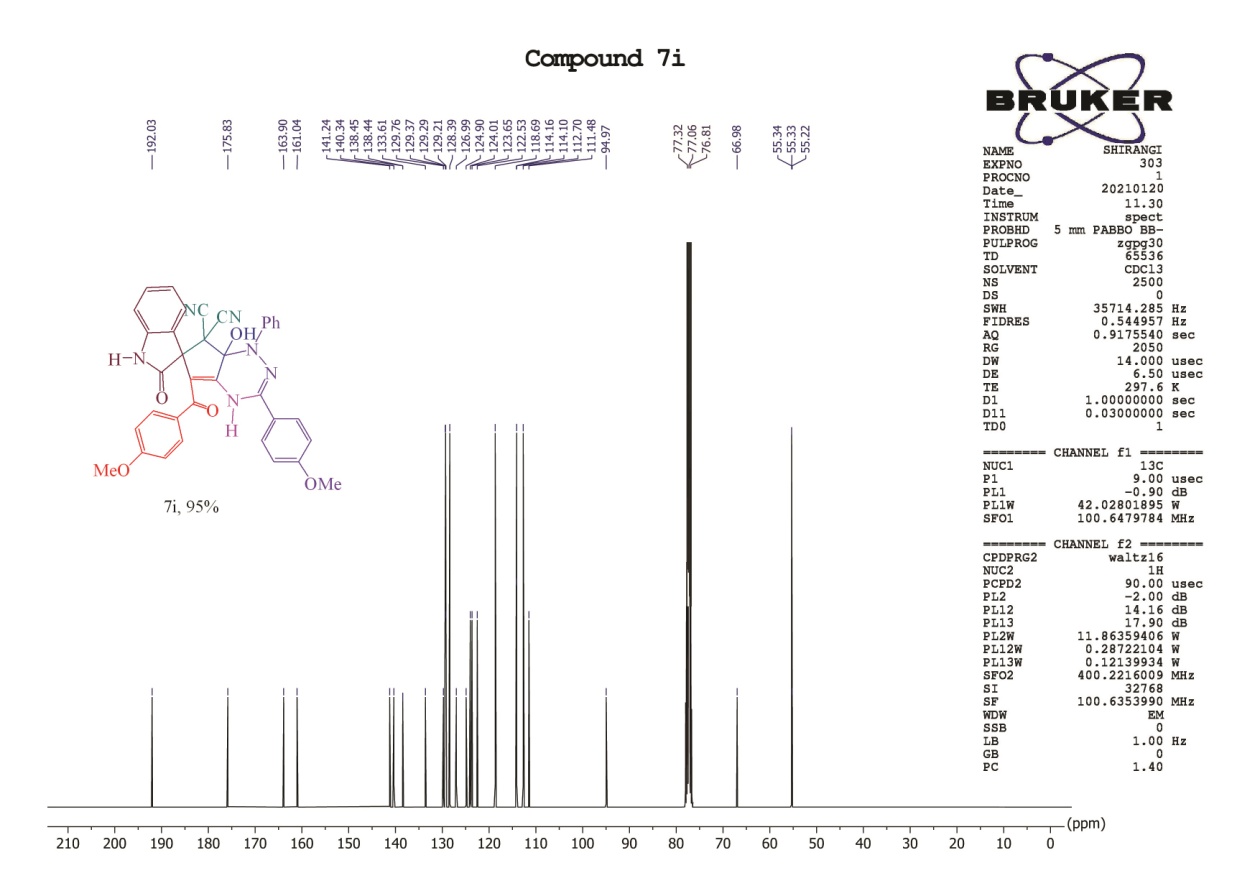


^13^CNMR Compound **7i**

**
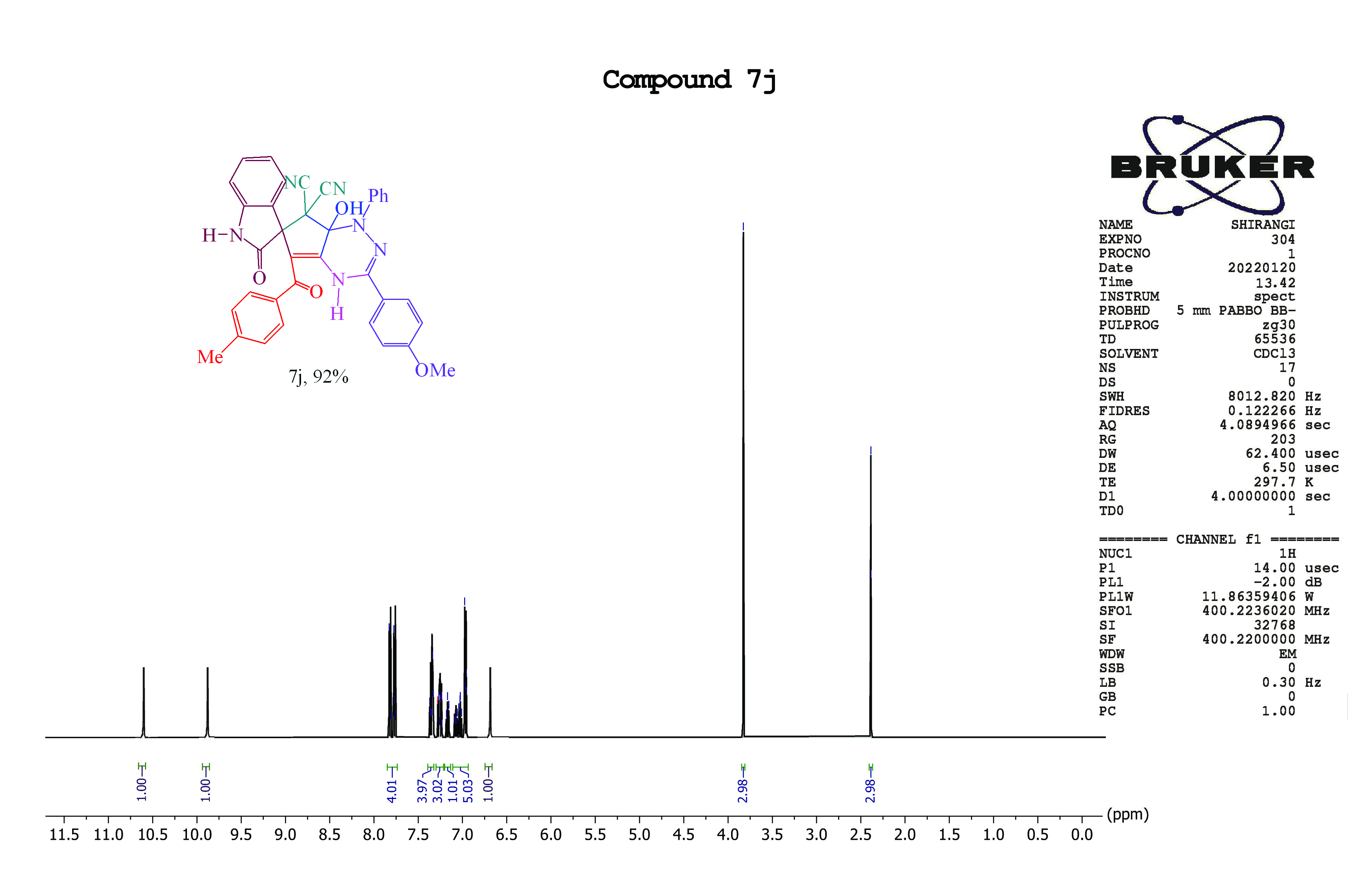
**

^1^HNMR Compound **7j**

**
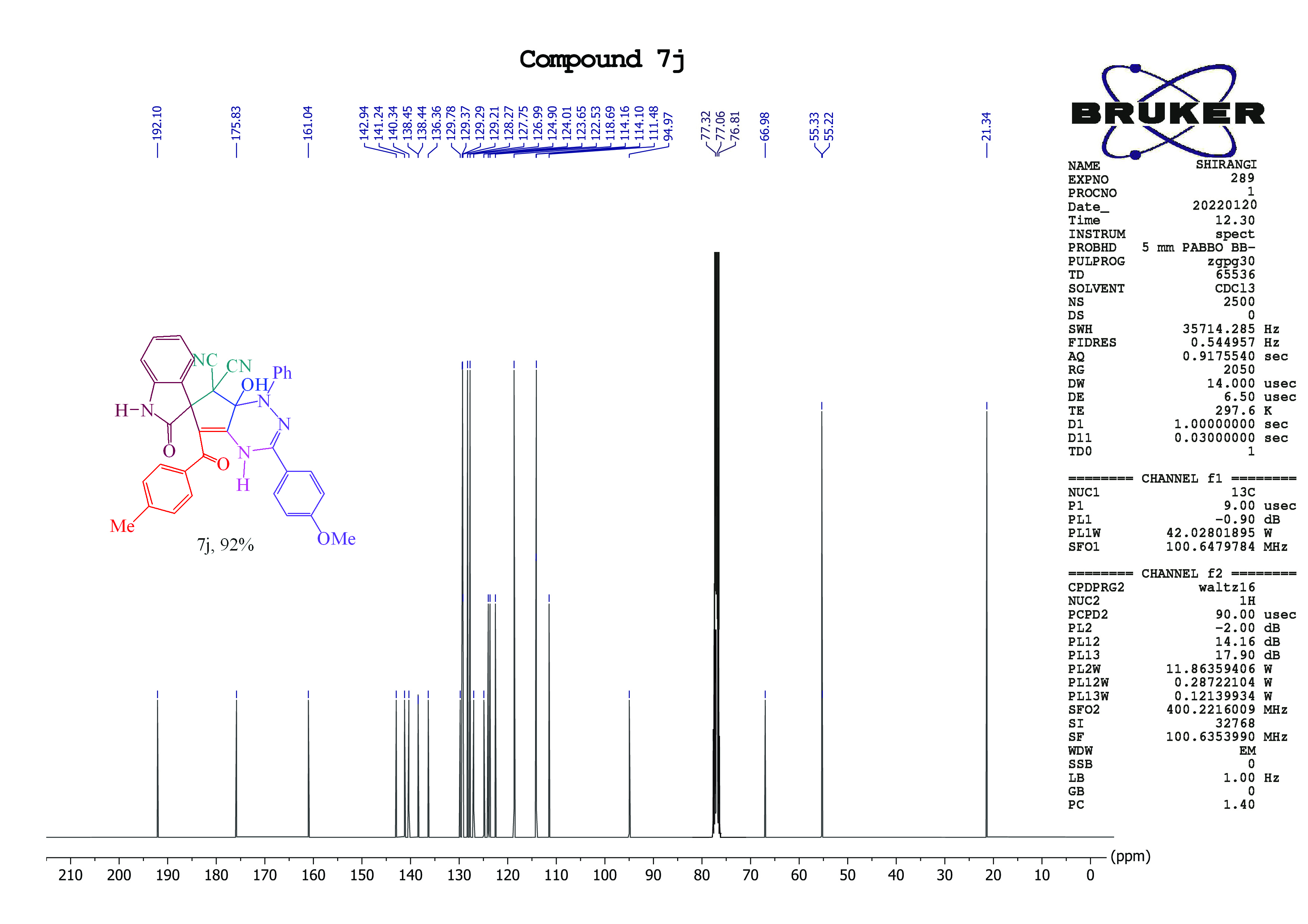
**

^13^CNMR Compound **7j**

**
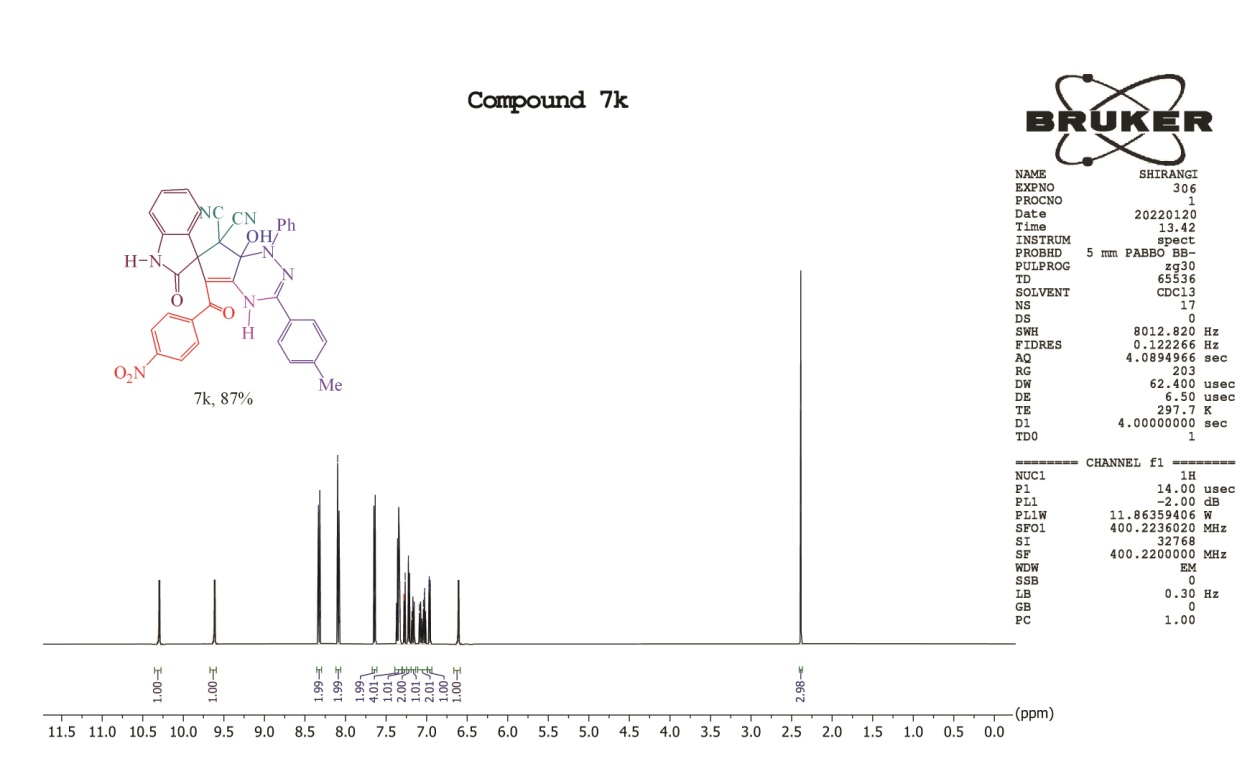
**

^1^HNMR Compound **7k**

**
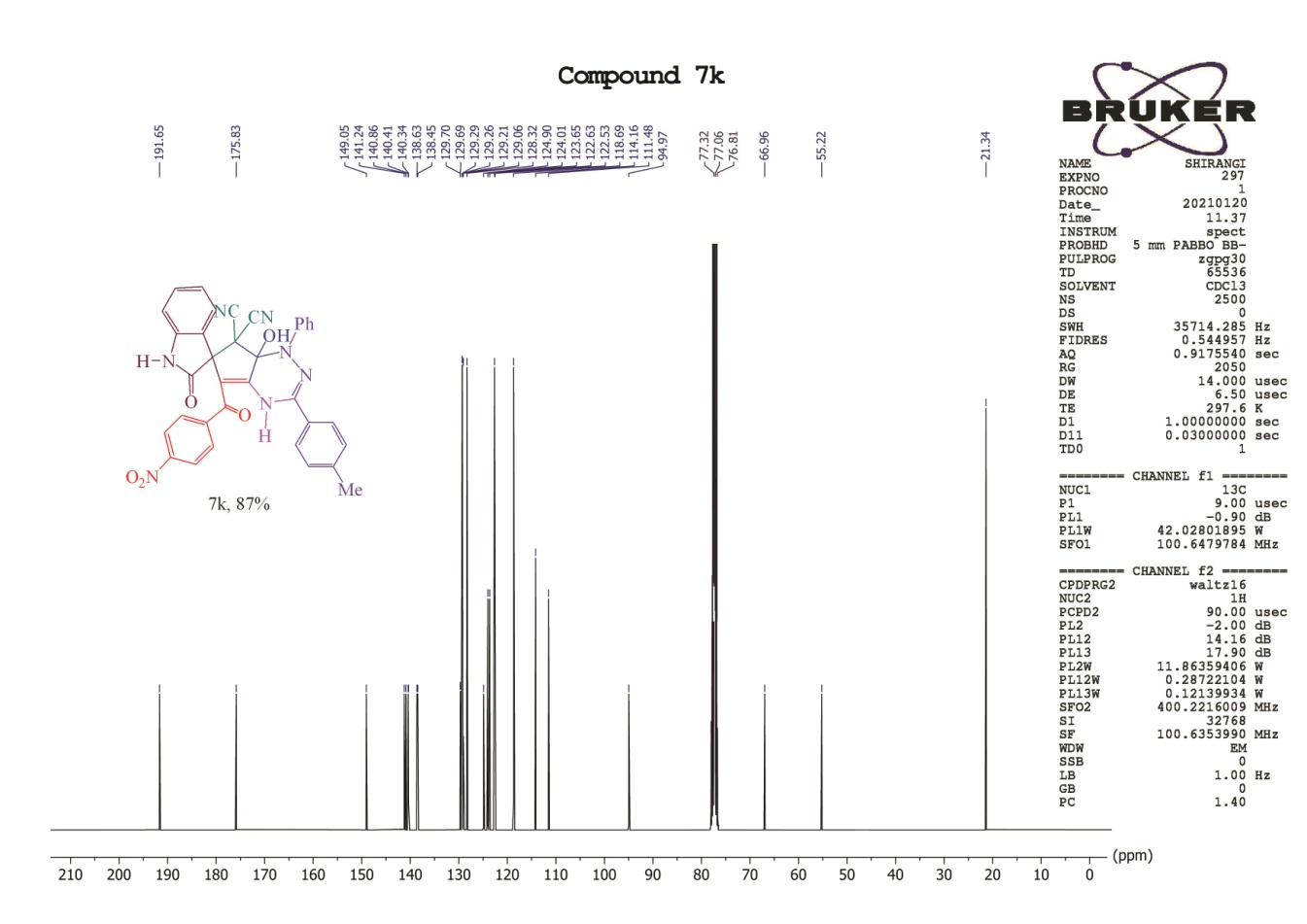
**

^13^CNMR Compound **7k**

**
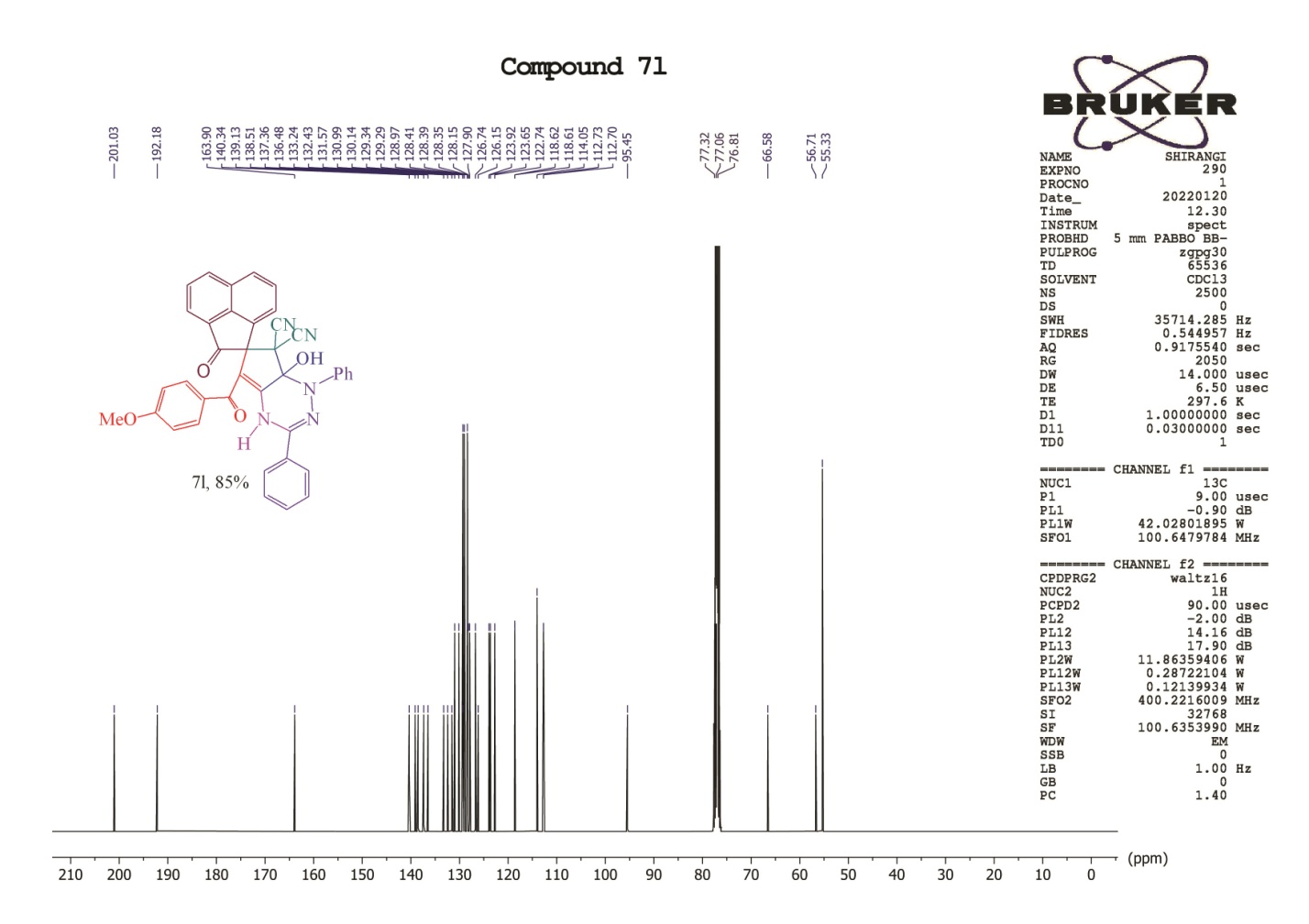
**

^13^CNMR Compound **7l**

**
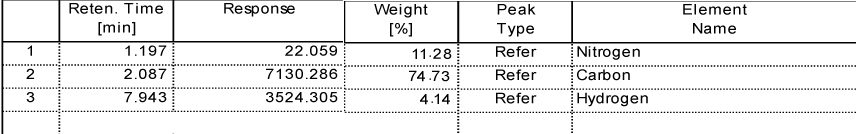
**

CHN of Compound **7l**

**
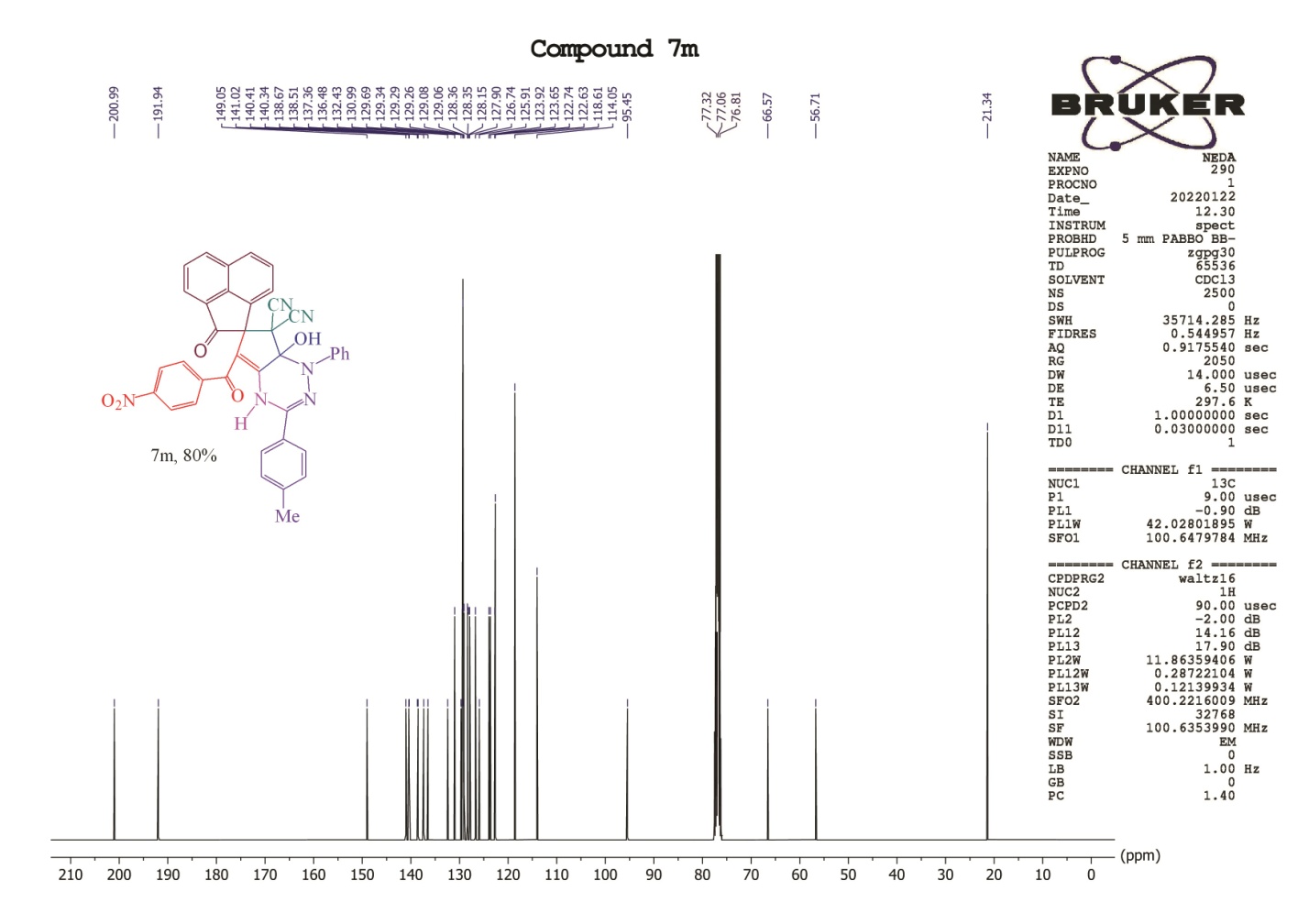
**

^13^CNMR Compound **7m**

**
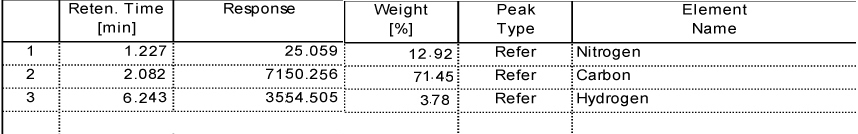
**

CHN of Compound **7m**

**
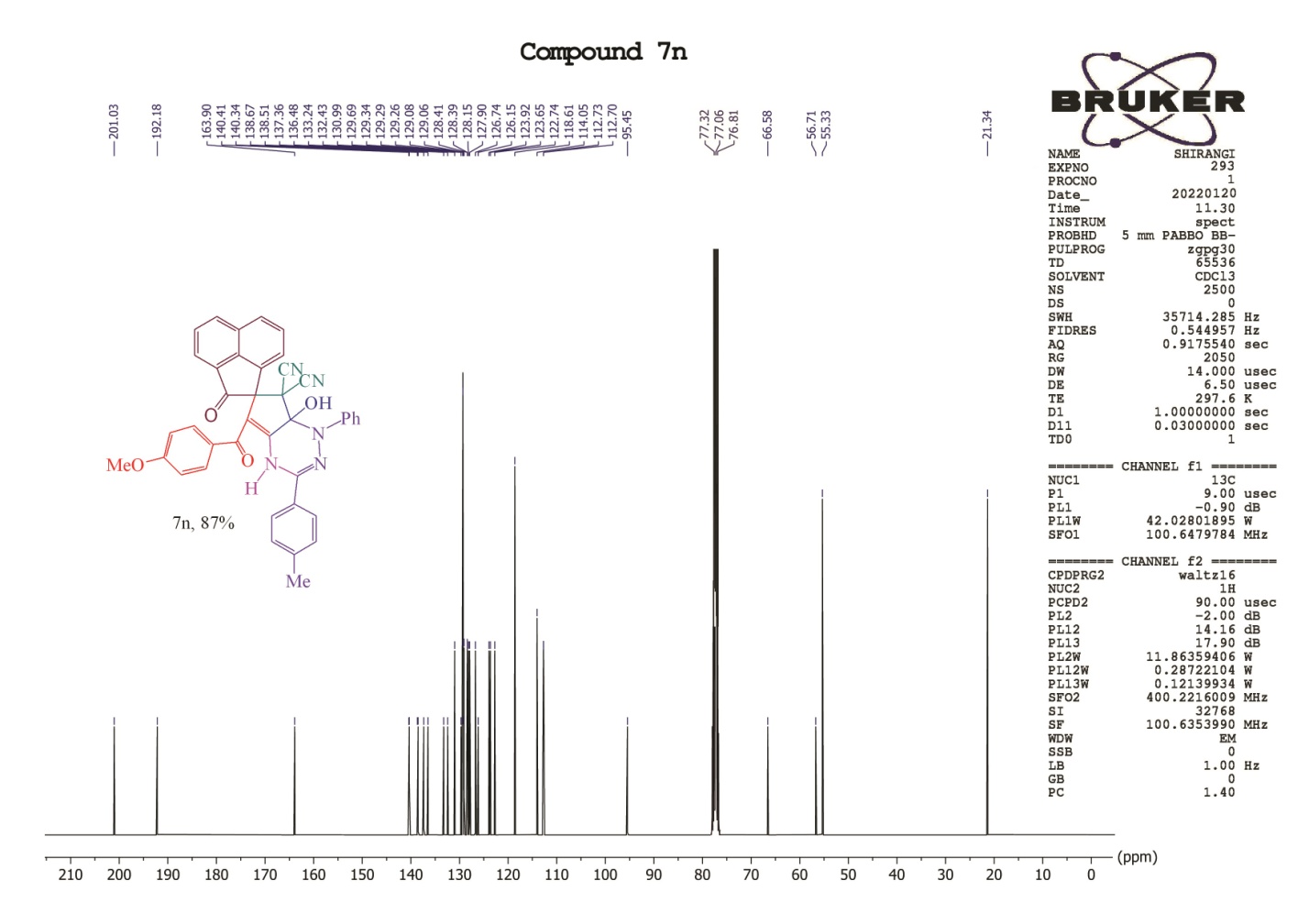
**

^13^CNMR Compound **7n**

Production of Ag NPs *via* water extract of *Petasites hybridus* rhizome


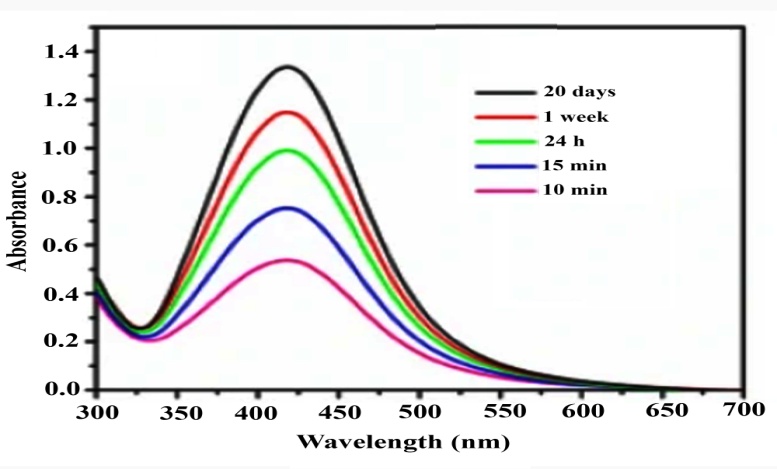


UV–Vis analysis of produced Ag NPs


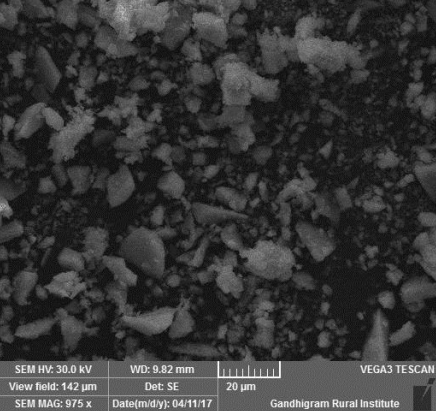

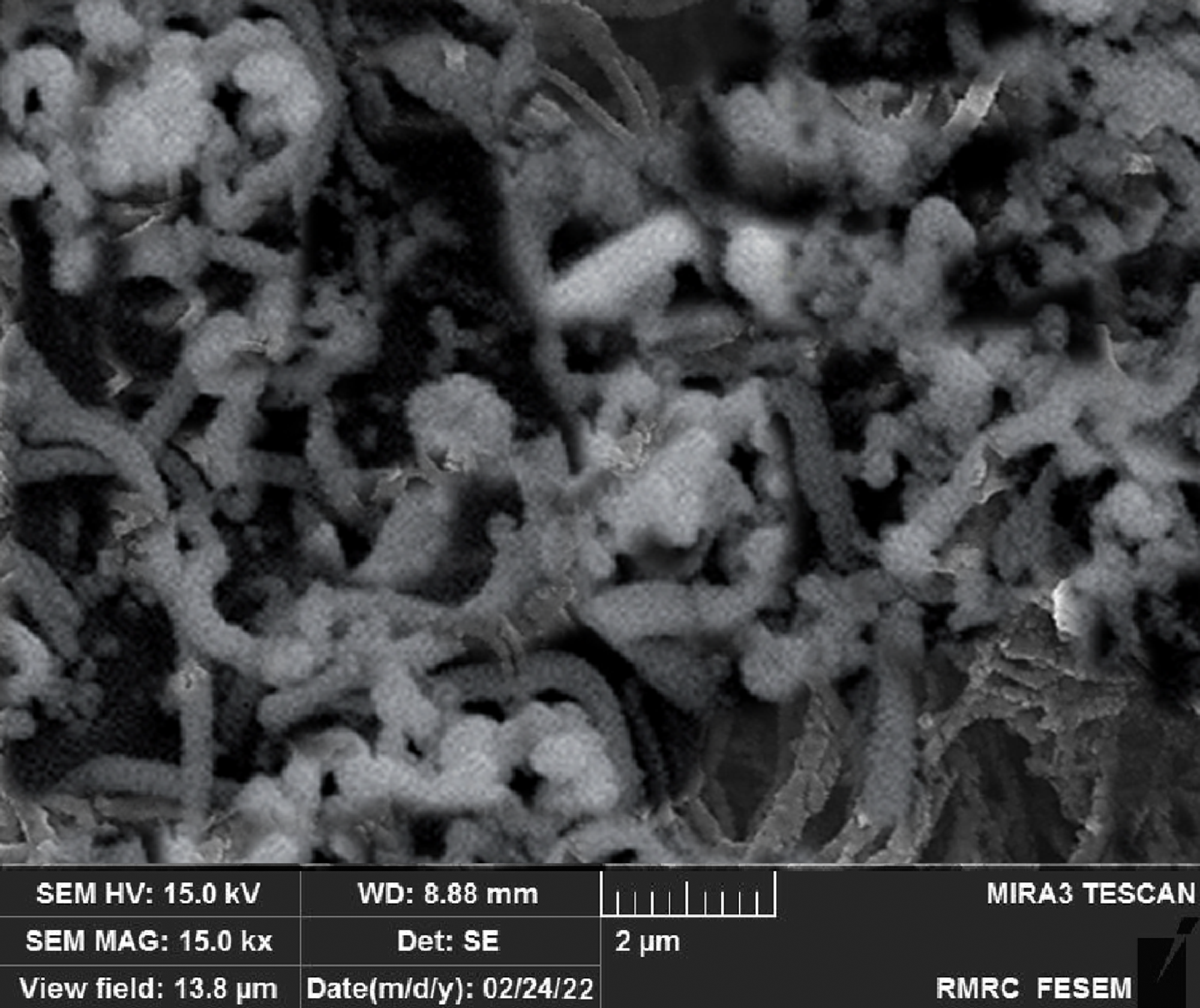


Left) the scanning electron microscopy image of Ag/Fe_3_O_4_/CdO right) SEM image of Ag/Fe_3_O_4_/CdO@MWCNT MNCs

**
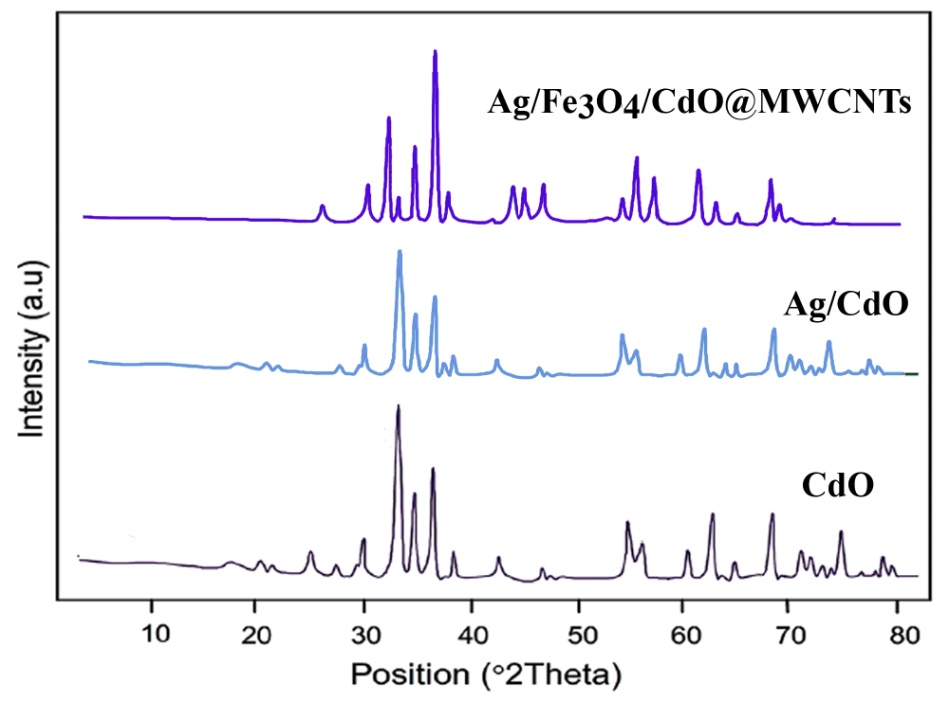
**

The XRD analysis of Ag/Fe_3_O_4_/CdO@MWCNT MNCs


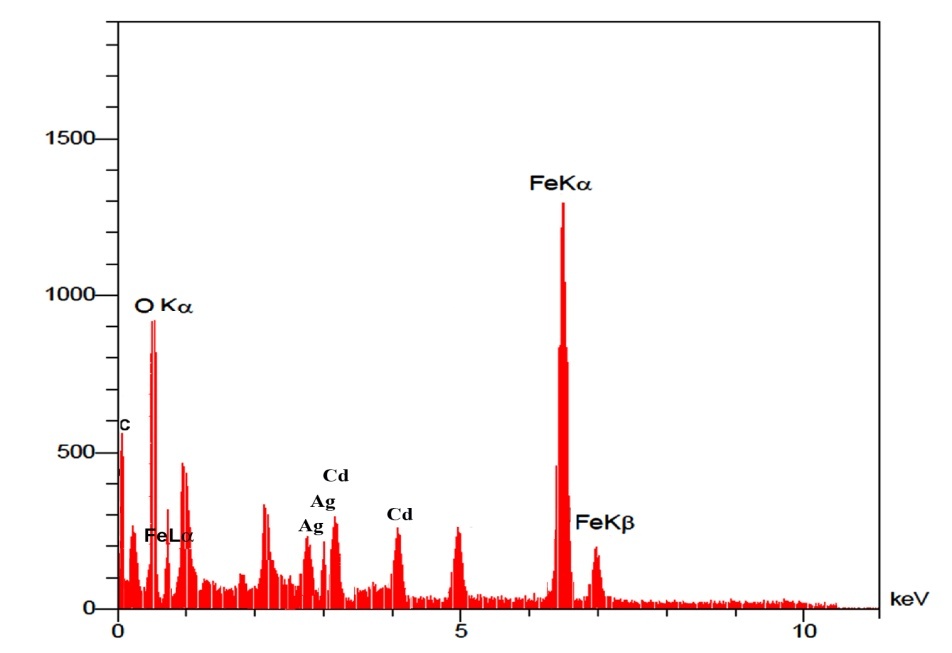


EDX image of Ag/Fe_3_O_4_/CdO@MWCNT MNCs


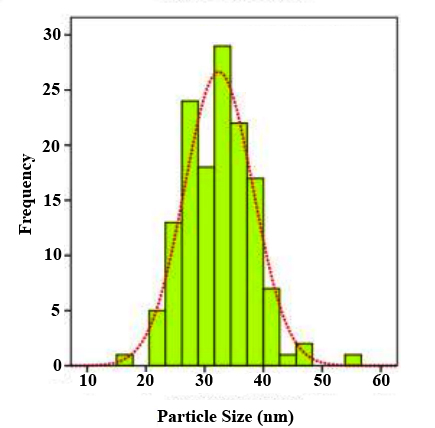

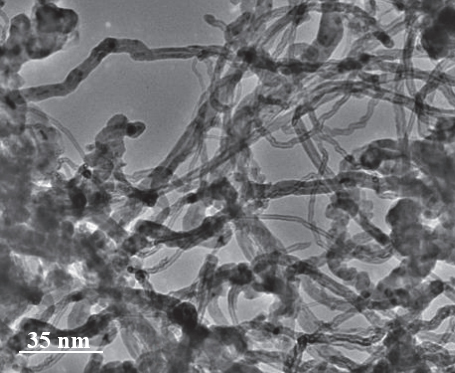


TEM image and histogram curve Ag/Fe_3_O_4_/CdO@MWCNT MNCs


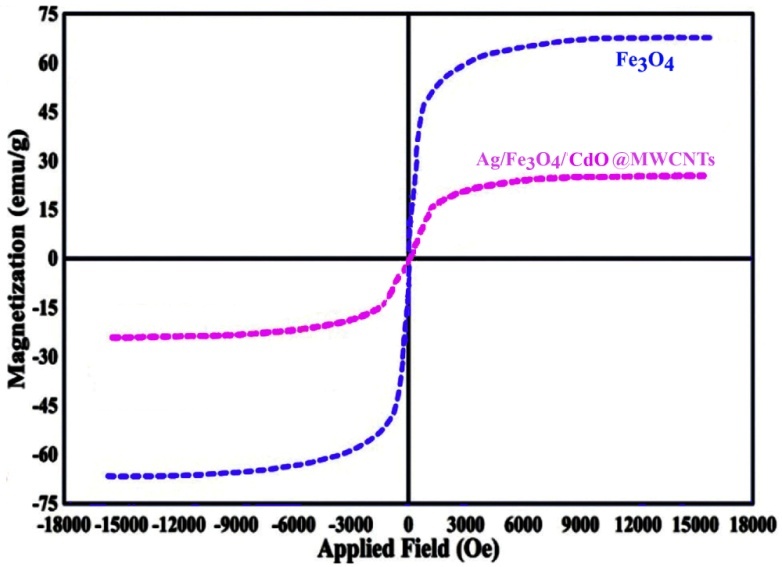


VSM analysis of the green Ag/Fe_3_O_4_/CdO@MWCNT MNCs


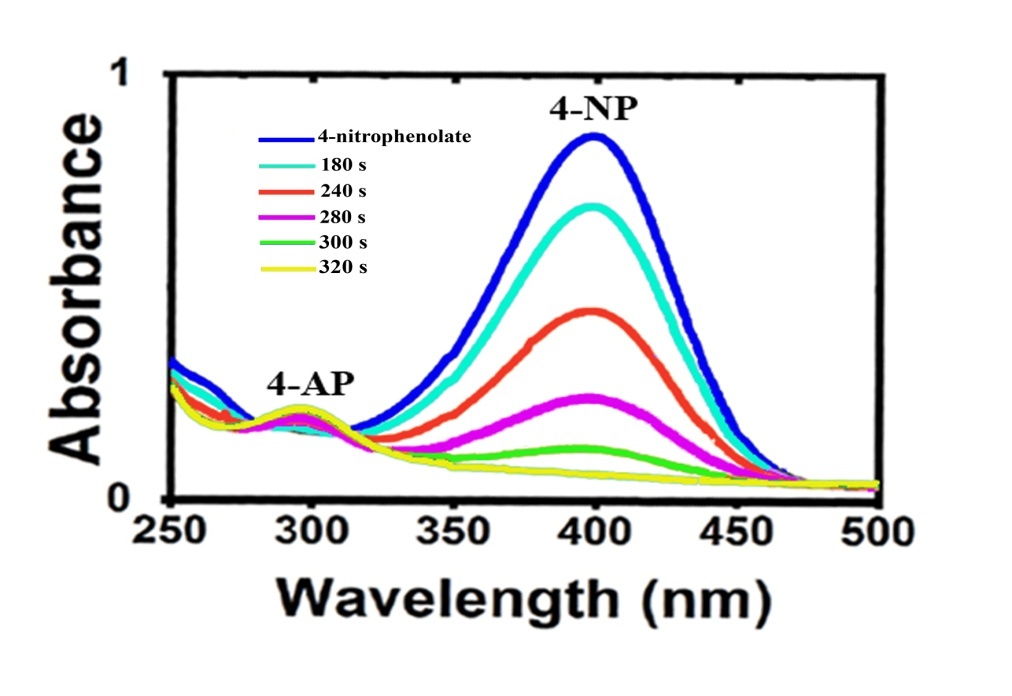


Reduction of the 4-NP to 4-AP by UV–Vis analysis
